# Comparison of Matrix Product State and Multiconfiguration Time-Dependent Hartree Methods for Nonadiabatic Dynamics of Exciton Dissociation

**DOI:** 10.1021/acs.jctc.4c00751

**Published:** 2024-10-04

**Authors:** Maximilian F. X. Dorfner, Dominik Brey, Irene Burghardt, Frank Ortmann

**Affiliations:** † TUM School of Natural Sciences, Technische Universität München, 85748 Garching bei München, Germany; ‡ Institut für Physikalische und Theoretische Chemie, Goethe Universität Frankfurt, 60438 Frankfurt am Main, Germany

## Abstract

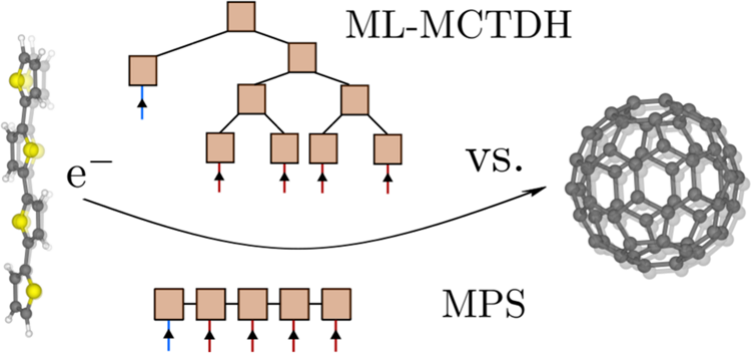

The excited-state dynamics of organic molecules, molecular aggregates, and donor–acceptor clusters is typically governed by the interplay of electronic excitations and, due to their flexibility and soft bonding, by the interaction with their vibrations. This interaction in these systems can be characterized by a few relevant electronic states that are coupled to numerous vibrational normal modes, encompassing a vast configurational space of the molecules. The full quantum simulation of these type of systems has been long dominated by the multiconfiguration time-dependent Hartree (MCTDH) approach and its multilayer variants, which are considered the gold standard in the presence of electron-vibration coupling with a large number of modes. Recently, also the matrix product state ansatz (MPS) with appropriate time-evolution schemes has been applied to these types of Hamiltonians. In this article, we provide a numerical comparison of excited-state dynamics between the MCTDH and MPS approaches for two electron-vibration coupled systems. Notably, we consider two models for exciton dissociation at a P3HT:PCBM heterojunction, comprising two electronic states and 100 vibrational modes, and 26 electronic states and 113 vibrational modes, respectively. While both methods agree very well for the first model, more pronounced deviations are found for the second model. We trace back the divergence between the methods to the different way entanglement is treated.

## 1 Introduction

Investigations of interacting quantum systems typically involve high-dimensional mathematical representations. As a matter of fact, such interactions are present everywhere from single molecules to solid state materials and this so-called “curse of dimensionality” is a major challenge for describing the dynamical properties of such materials. To overcome this challenge in the context of electron-vibration coupled molecular systems, the multiconfiguration time-dependent Hartree (MCTDH) method^1,2^ and its multilayer variants (ML-MCTDH)^3−5^ have been developed as pivotal advancements, exploiting low-rank tensor approximations. Specifically, ML-MCTDH allows addressing this challenge by systematically decomposing the full product space of electronic states and vibrational modes into a tree-like, tensorial structure, a tree tensor network. Such decomposition, together with a focus only on the relevant states in an otherwise exponentially scaling Hilbert space, makes the problem tractable and enhances computational efficiency.

This effectiveness of MCTDH and ML-MCTDH have quickly set a new standard in the field, particularly for the description of nonadiabatic effects in the photophysics of organic molecular species in the gas phase, which typically exhibit ultrafast internal conversion induced by conical intersections.^6,7^ A prototypical case is the exploration of pyrazine’s unusual photophysics, which had gathered significant attention among theoretical chemists^6,8−14^ because of its interesting *S*_1_ – *S*_2_ conical intersection in the near ultraviolet (UV) region. The involved states are nonradiatively coupled through a conical intersection,^15^ which leads to an unexpectedly broad absorption band, due to dynamical mixing of both states, involving vibrational modes. Early theoretical investigations,^8−10^ including few relevant modes, could qualitatively reproduce the absorption spectrum confirming the origin of the broad band. Moreover, it was the work of refs (13,16,17) taking into account all 24 vibrational modes and second order nonlinearities of the potential energy surface that cleared up any doubts about the origin of the *S*_1_ – *S*_2_ absorption band and contributed to positioning these methodologies as reliable tools in modern quantum chemistry. Today’s state-of-the-art calculations based on ML-MCTDH involve up to hundreds of electronic states and vibrational modes.

Largely in parallel to these developments, the theoretical condensed matter community has developed the matrix product state (MPS) approach, another tensor network method, to address similar high-dimensional challenges in quantum physics. In order to represent quantum states, the MPS form has an underlying one-dimensional tensor network structure,^18^ which has been shown to be particularly well suited to represent the ground state of gapped one-dimensional systems.^19^ While its origins lie in the ground-state search of paradigmatic one-dimensional model systems using the density-matrix renormalization group method (DMRG),^20,21^ subsequent extensions of the MPS methodology, pioneered by refs (22−24), have removed these initial limitations and enabled the computation of time evolution in this framework. Subsequently, the representation of thermal states was elaborated,^25^ further extending the capabilities of MPS-based methods.

The MPS methodology has also been applied to problems from quantum chemistry. Examples include the use of the DMRG algorithm for ground-state search of molecules,^26,27^ the calculation of vibrational eigenstates of molecules,^28^ or the computation of nonadiabatic dynamics^29−31^ of coupled electron-vibrational systems. This contributed to the mutual awareness of different methods across different communities and naturally raises the question whether the MPS and ML-MCTDH methodologies can be considered essentially equal or even fully equivalent for computing the nonadiabatic dynamics, or if differences can be observed for typical model systems. For the pyrazine-based two-level model with up to 24 modes^13,32^ and the perylene bisimide trimer model^33−37^ with four modes per molecule, both methods agree. While the agreement for these models is encouraging, these comparisons are still rather limited in terms of the number of involved modes and electronic states. Much less is known about how both frameworks compare for larger models representing spatially extended systems, both for a larger number of electronic states and for a larger number of modes that are typically found in more application-relevant situations such as interfaces in organic photovoltaics (OPV). This article aims to contribute to the knowledge in this direction, by providing a numerical comparison between the standard formulations of the ML-MCTDH and the MPS approach for two models that fall into this class.

We find that MPS and ML-MCTDH treat the first, less strongly entangled model with only minor differences, whereas for the more complex model, significant quantitative differences emerge after some time. Notwithstanding, a consistent qualitative physical picture is obtained by both methods. Furthermore, in both models, deviations in the entanglement entropy suggest differences in how entanglement between the electronic and vibrational degrees of freedom is treated in MPS and ML-MCTDH, possibly due to the different network structure.

## 2 Models and Methods

### 2.1 Model

While the radiationless transitions induced by conical intersections remain an interesting topic even for isolated molecular species, there are other, technologically more important cases where the same type of physics is important. One of these relevant examples is the charge separation process at organic donor–acceptor interfaces in bulk heterojunctions, important for the power conversion efficiency in organic solar cells.^38^ There, a tightly bound Frenkel exciton, located at the donor molecule at the interface, is separated into a less strongly bound charge-transfer exciton, consisting of a hole and electron localized on either donor and acceptor molecules. This process is not only influenced by electronic states but also by the vibrational modes of the involved molecules.^39,40^ In the following, we are interested in the exciton dynamics at the interface of an *n*-oligothiophene (OT_*n*_) donor—C_60_ fullerene acceptor system, which can be regarded as a simplified structure model of the prototypical bulk heterojunction material blend poly-3-hexylthiophene (P3HT) and phenyl-C_61_ butyric acid methyl ester (PCBM).^41^

In order to describe the interplay of electronic and vibrational states at the interface affecting the charge separation process, we restrict ourselves to a linear-vibronic coupling model^6,15^ of the general form
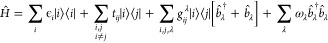
1where, *t*_*ij*_ are the transfer integrals, and ϵ_*i*_ denote the on-site energies of the electronic states |*i*⟩ respectively |*j*⟩. Furthermore, *b̂*_λ_^†^ respectively *b̂*_λ_ denote the creation and annihilation operator of vibrational mode λ with energy quantum ω_λ_ and *g*_*ij*_^λ^ the linear coupling constant.

#### 2.1.1 Model A

**Figure 1:**
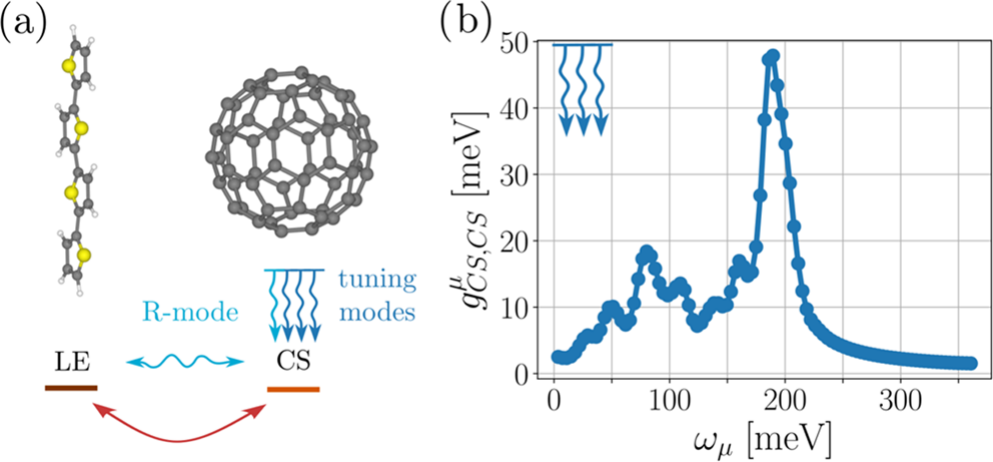
(a) Visualization of the electronic part and coupling structure of model A. Lines indicate the respective electronic on-site energies, whereas the arches denote the transfer integrals *t*_*ij*_. The wiggly lines indicate the coupling structure of the modes. All energies in meV. (b) Coupling strength *g*_CS,CS_^μ^ plotted against mode frequency ω_μ_ for the 99 tuning modes used in the calculation.

A minimal model to describe the charge separation process only involves one donor and one acceptor site and therefore only one local-exciton state (LE) and one charge-transfer state (CS), but a large number of modes. Here, we consider the OT_4_ – C_60_ system as described and parametrized in ref (42) and additionally studied, in different variations, in the refs (43−45). Let us therefore only briefly summarize the main points of this model, the Hamiltonian is specified in the Supporting Information (Section S2). This model involves two electronic states |LE⟩ and |CS⟩. These are electronically coupled by a transfer integral *t*_LE,CS_ = 130 meV and the CS state lies 79 meV below the LE state (ϵ_CS_ = −79 meV). This electronic system is coupled to a single intermolecular mode (ω_R_ = 10 meV), which dynamically couples the two electronic states (*g*_LE,CS_^R^ = −10/ meV) and, at the same time, tunes the energy of the CS state (*g*_CS,CS_^R^ =  meV) upon displacement (see ref (42) for discussion). Furthermore, there are 99 discretized tuning modes, labeled by μ, which modulate the CS state energy *g*_CS,CS_^μ^ ≠ 0. They are generated by effective mode reduction from the full normal-mode space^43^ and their specific values are taken from ref (44). We illustrate the interaction between the electronic states and the modes in Figure 1(a) and plot the tuning mode energies and their respective coupling strength in Figure 1(b). For reproducibility, the data underlying this plot can be found in Table S1 and Section S2 of the Supporting Information. This model, which includes 100 nontrivially coupled vibrational modes, will serve as our initial testing ground for comparing the MPS and ML-MCTDH methodologies.

#### 2.1.2 Model B

**Figure 2:**
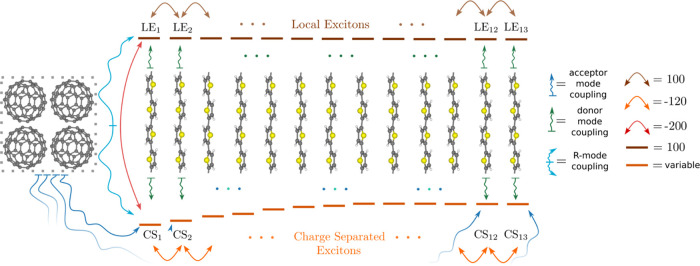
Visualization of the electronic and vibrational coupling structure of model B. Lines indicate the on-site energies of the respective electronic states and arrows denote the transfer integrals between the electronic states. The on-site energies of the CS_*n*_ states constitute an effective Coulomb barrier. The fullerene particle represents an effective coarse-grained acceptor site.^46^ Wiggly lines indicate vibrational coupling between the modes of the fragment (or the R-mode) and the electronic states. All energies are given in meV.

While model A is able to describe the initial charge separation at the donor–acceptor interface, it does not account for the long-range charge separation under the influence of an effective Coulomb barrier in organic materials. Due to this long-range interaction, it is usually not only the interfacial moieties that influence the charge separation process, but a manifold of other excitonic and charge-separated states that are present. To account for this, we next consider an electronically extended system, which is illustrated in Figure 2. In this model not only one OT molecule is present next to the C_60_ domain but an array of 13, cofacially stacked oligothiophenes, mimicking one direction of a regioregular OT-rich phase at the donor–acceptor interface. This model has also been used in refs (46−48), where different effective Coulomb barriers were constructed, depending on the degree of electron delocalization across the fullerene domain, which is here represented as an effective coarse-grained acceptor site. A generalized electron–hole representation is used, where we account for 13 local-exciton states (LE_1_, ..., LE_13_), in which hole and electron are localized at the same OT donor site, and 13 charge-separated states (CS_1_, ..., CS_13_), where the electron is localized at the acceptor site and the hole is localized at the respective OT site. Thus, this model contains 26 electronic states in total. In this representation, the electronic part of the Hamiltonian is tridiagonal, see the explicit form of the Hamiltonian given in the Supporting Information (S3). The number of normal modes of the C_60_ and the OT fragments is reduced by an effective-mode procedure,^46^ such that 8 effective modes for the fullerene acceptor site and 8 modes for each of the OT molecules are taken into account. Additionally, the intermolecular mode, describing the relative motion of the C_60_ and the first OT molecule (cf. model A) is taken into account. This amounts to 113 vibrational modes in total. The LE states couple naturally only to the modes of the OT molecule, where they are localized. In contrast, the CS_*n*_ states couple to the C_60_ modes and to the modes at the *n*th OT molecule, as the electron is located at C_60_ and the hole is at the *n*th OT molecule. The intermolecular mode couples only to the LE_1_ state and the CS_1_ state. This coupling is also illustrated in Figure 2. There the electronic matrix elements can be found, whereas the CS_*n*_ on-site energies, the coarse-grained mode energies, and their coupling constants are summarized in the Supporting Information (Section S3, and Tables S2 and S3).

#### 2.1.3 Initial State

In the following, we are interested in the Hamiltonian dynamics of a given initial state |ψ_0_⟩, which we assume to be a product state between the electronic and vibrational degrees of freedom, in accordance with the Franck–Condon principle. We further assume that the initial state is located right at the interface. This means that the initial state is given by
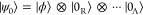
2where, |0_λ_⟩ denotes the ground state of the harmonic oscillator labeled by λ, |ϕ⟩ = |LE⟩ for model A, and |ϕ⟩ = |LE_1_⟩ for model B.

### 2.2 Tensor Network Methods for Nonadiabatic Dynamics

Both the MPS and ML-MCTDH methods fall into the general class of tensor network methods^49^ which rely on a suitable ansatz for multidimensional wave functions in conjunction with the time-dependent variational principle (TDVP).^50−54^ The TDVP yields a projected version of the Schrödinger equation (SE),
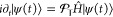
3where,  denotes the projector onto the tangent space, i.e., the linear space spanned by the permitted wave function variations.^50^ For a wave function ansatz comprising multiple electronic states and vibrational modes, the following general sum-of-products form
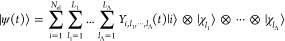
4with time-independent basis functions |χ_*l*_κ__⟩ is reduced to a low-rank tensor approximation by recasting the coefficient tensor *Y*_*i*,*l*_1_,···,*l*_Λ__ in a suitable tensor format. For example, the so-called Tucker format underlies the MCTDH method,^55^
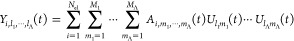
5where, *A*_*i*,*m*_1_,···,*m*_Λ__ is the core tensor, *U*_*l*_κ_*m*_κ__ are transformation matrices, and *M*_κ_ ≪ *L*_κ_ with κ ∈ {1, ···, Λ} such that a compact representation is obtained. A generalized, hierarchical Tucker format corresponds to the ML-MCTDH method^4,5,56,57^ as detailed below. Matrix product states entail yet another reduction of the above core tensor, which we discuss in more detail in the upcoming subsection.

**Figure 3:**
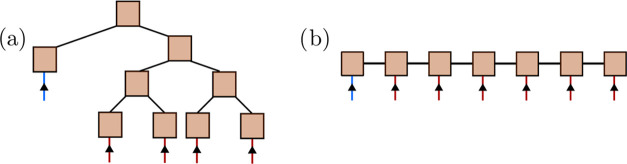
Schematic illustration of the two different tensor network structures used to represent the many-particle states in this study. (a) Tree tensor network of the ML-MCTDH method; (b) chain-like tensor representation of the MPS approach. The squares indicate the respective tensors. The blue arrows stand for the electronic indices, the red arrows represent the harmonic oscillator indices of the modes, and connecting black lines indicate a contraction of the corresponding tensors.

Hierarchical tensor networks of the ML-MCTDH type are also termed tree tensor networks.^58,59^ ML-MCTDH schemes then correspond to balanced trees of minimal height, while MPS schemes correspond to sparse trees of maximal height. The latter is alternatively termed tensor trains. Obviously these construction schemes exhibit different approaches toward accommodating temporal and spatial correlations, and, hence, they may exhibit different convergence properties.^59,60^ The different network structures underlying the ML-MCTDH and MPS approach are schematically depicted in Figure 3. In the following, we provide further details on the MPS and ML-MCTDH methods and their implementation.

#### 2.2.1 Matrix Product States for Nonadiabatic Dynamics

We first discuss the nonadiabatic dynamics of an initial state |ψ_0_⟩ in the MPS representation of states in the Hilbert space of the Hamiltonian eq 1 with the ansatz
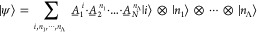
6Here, *i* labels the electronic basis states, whereas *n*_λ_ indicates the *n*th eigenstate of the harmonic oscillator λ (1 ≤ λ ≤ Λ) and *A*_*k*_^μ^ are matrices of appropriate size for the matrix multiplication to be well-defined. The index *k* ∈ {1, ···, *N*} labels the respective factor space, whereas μ ∈ {*i*, *n*_1_, ···, *n*_Λ_} is associated with the basis state index in the corresponding factor space. The ordering chosen in eq 6, setting the electronic factor space at the left end of the MPS chain, is fixed throughout this study. The arrangement of eq 6 is useful when considering a limited number of relevant exciton states. In cases where the full electronic Fock space is relevant, e.g., for multiexciton processes, a different arrangement of the individual factor spaces might be more appropriate.^36^ Alternatively, so-called “multi-set” representations, where state-specific nuclear wave functions are introduced, are conceivable options.^61,62^ The remaining freedom of ordering the nuclear degrees of freedom is discussed specifically for model A and model B.

The size of the matrices *A*_*k*_^μ^ determines the quality of approximation of the state. It is called bond dimension or link dimension *D* and can in principle be increased to obtain the exact solution. In any practical calculation, however, one has to choose a finite, but sufficiently large bond dimension to obtain converged results. Additionally to the approximation of using only a limited bond dimension in any numerical implementation, the used basis states for the bosonic degrees of freedom have to be truncated, like in ML-MCTDH.

Apart from an MPS to represent the state in the Hilbert space, a representation of operators—so-called matrix product operator representation (MPO)—of the Hamiltonian eq 1 is required. Thus, extending the MPS representation to MPOs, one can cast the Hamiltonian in the form
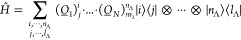
7where (*O*_*k*_)_ν_^μ^ are matrices of appropriate size, with the notation introduced before. Naively, this MPO can be constructed by summing up individual product operator contributions. However, this procedure naturally increases the bond dimension of the MPO with every term added with the sum of the individual bond dimensions.^18^ To circumvent this, we perform subsequent compressions^63,64^ of the individual terms to obtain the respective MPO representation.

The initial product state |ψ_0_⟩ of the model, by construction, admits an exact MPS form of bond dimension one. The time evolution driven by the Hamiltonian eq 1 leads to a growth in entanglement between the electronic and vibrational system, which requires a larger bond dimension of the time-evolved MPS. Referring to the projected Schrödinger equation of eq 3 in the case of the MPS approach, the symbol  denotes the projector onto the linear space spanned by the states where one or two tensor *A*_*k*_^μ^ in the MPS is allowed to be varied, which constitute the tangent space of the MPS. This projected version of the SE is then approximately solved by sequentially time-evolving each of the individual tensors *A*_*k*_^μ^(*t*) (1TDVP), or a single contraction of them (2TDVP), with respect to an effective Hamiltonian, determined by the other tensors at this time step. This approach transforms the global problem of evolving a state in the full Hilbert space into a sequence of local problems, only involving the local tensors in effective environments. In contrast to 1TDVP, the 2TDVP allows for a dynamic increase of the bond dimension *D*, during the time evolution.

In principle, there are four sources of error compared to the exact solution:^54^ The projection error, a time step error, an error related to the inexact solution of the local problem, and a truncation error, which is only present in the 2TDVP version. In most cases, the dominant one is the projection error. This error can be traced back to solving the projected version of the SE, and not the full one, and can be reduced by enlarging the bond dimension *D*, which allows a systematic check of convergence. For chain-like geometries with (semilocal) interaction, the 2-site version is regarded as the most reliable algorithm for the time evolution of an MPS.^54^ Still, there are situations where the original form of either algorithm shows limitations. This turns out to be the case, e.g., when the initial state is a product state for Hamiltonians with long-range interaction.^65^ Beyond the TDVP algorithms, there are also other time-evolution algorithms for the computation of the nonadiabatic dynamics in the MPS framework.^66−69^

For all of the MPS-related work in this study, we used the Itensor library.^70^ Unless stated otherwise, we use a compression threshold in the MPO construction of 10^–13^ for the kept singular values. We estimated the necessary number of oscillator states for a mode λ, by
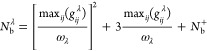
8where the first term corresponds to the mean in the case of localized electronic levels coupled to vibrations (static polaron case),^71^ the second term is 3 times the standard deviation of the value in the static polaron case and *N*_b_^+^ is a numerical offset to check for convergence. If more excitations are necessary, one can restore the U(1) symmetry of the phonons at the cost of doubling the factor spaces of the phonons, which is called projected purification.^72^ However, at least in some cases, this may not be necessary, for example the exciton dynamics of the singlet fission in rubrene^29^ can be successfully described with only 10 vibrational quanta per mode.^73^ For the time evolution, we use the TDVP code as implemented and used in ref (65) and publicly available at^1^. At initial times, we use a global Runge–Kutta integrator (RK), to enlarge the bond dimension of the initial product state to circumvent a trapping in the low-entanglement sector. As this approach is at some point computationally prohibitively expensive, we switch to the 2TDVP method to further increase the bond dimension. However, the significantly worse execution-time scaling of 2TDVP compared to 1TDVP limits the accessible time scales with 2TDVP. For this reason, we only use 2TDVP to enlarge the bond dimension up to a certain maximum bond dimension *D*_max_ and perform time evolution from this time on using 1TDVP. It should be noted that other schemes to extend the bond dimension for 1TDVP exist.^37,65,74^ In this regard, the combination RK-2TDVP is a possible choice to dynamically increase the bond dimension of the MPS. This procedure has been tested successfully for a smaller but similar model compared to the ones presented here [cf. Supporting Information Section S1] against the RK method.

#### 2.2.2 Multilayer Multiconfiguration Time-Dependent Hartree Method

The ML-MCTDH method^4,5,56,57^ employed in the present work derives from the parent MCTDH method which relies on the Tucker format^75−77^ of eq 5, as mentioned in Section 2.2. The latter equation for the coefficient tensor *Y*_*i*,*l*_1_,···,*l*_Λ__ can alternatively be expressed as the inner product
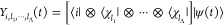
9for the MCTDH wave function |ψ(*t*)⟩
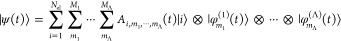
10where time-dependent auxiliary quantities denoted as single-particle functions (SPFs) |φ_*m*_λ__^(λ)^(*t*)⟩ were introduced whose inner product with the time-independent basis functions is given by *U*_*l*_λ_*m*_λ__(*t*) = ⟨χ_*l*_λ__|φ_*m*_λ__^(λ)^⟩, leading to eq 5.

In ML-MCTDH, the same construction scheme is used within a hierarchical Tucker format.^75−77^ That is, a first layer is constructed analogously to eq 10, but involving a smaller number (*K*_1_ < Λ) of higher-dimensional subspaces (each grouping *M*_κ_1__^(1)^ factor spaces)
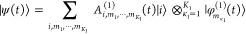
11Next, the first-layer SPFs comprising subsets of combined vibrational modes are expanded in second-layer quantities (*K*_2|κ_1__ ≤ *M*_κ_1__^(1)^), again according to the Tucker format
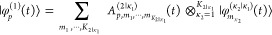
12Generally, the SPFs of the first *M* – 1 layers are given as follows
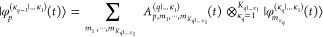
13where *q* = 2, 3, ..., *Q* runs over the layers and the SPF index κ_*q*_ = 1, 2, ..., *K*_*q*|...κ_1__ runs over the *q*th layer modes. Finally, the SPFs of the last (*Q*th) layer are represented in the time-independent basis |χ_*l*_λ__⟩. At this point, the expansion in the primitive basis is inexpensive since it is done in low-dimensional subspaces. The details of the hierarchical ML-MCTDH wave function form can be represented in terms of a multilayer tree.^49^ The structure is highly flexible and often used to construct balanced trees comprising different subtrees that can accommodate varying numbers of layers. In the present calculations, the construction of the multilayer trees is closely related to the structure of the physical system, which is composed of multiple donor and acceptor fragments. In the calculations for model B, tensor trees with up to *Q* = 8 layers were employed.

The equations of motion of the multilayer approach involve a hierarchy with the *q*th layer coefficients evolving under corresponding *q*th layer multiconfigurational mean fields. These equations are generally implemented using a recursive algorithm,^5,57^ as employed in the Heidelberg MCTDH package^78^ that was used in the present work. The time integration combines the integration of the time-dependent wave function coefficients with the integration of time-dependent SPFs. In the present study, the so-called variable mean-field (VMF) scheme was employed where the mean-field matrix elements are evaluated at every integrator step. The Adams-Bashforth-Moulton (ABM) predictor-corrector integration scheme was employed for both wave function coefficients and SPFs. Alternatively, in the so-called constant mean-field (CMF) integration scheme, one exploits that the coefficients and SPFs evolve on different time scales.^55^ In conjunction with the CMF scheme, the short iterative Lanczos (SIL) algorithm is typically used for the coefficient evolution, while the Bulirsch-Stoer (BS) integrator is used for the nonlinear equations of motion for the SPFs.^55,78^

Computation of the mean-field matrix elements is efficient if the Hamiltonian can be represented in a sum-of-products form, similarly to eq 7 in the case of MPS,
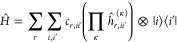
14This is naturally the case for model potentials of the type considered in this paper. In general, potential fitting algorithms can be employed to generate the desired sum-of-products form of the Hamiltonian; notably the so-called Potfit algorithm^55^ has been developed for this purpose, along with its adaptation to the ML-MCTDH method.^79^ Further, more general procedures like neural network potentials^80^ can be employed. Once a sum-of-products form is obtained, kinetic matrix elements are evaluated analytically using a polynomial basis set representation, and the associated discrete variable representation (DVR) is employed for the evaluation of potential matrix elements.^55^

Convergence of the calculations is monitored in terms of the time-evolving populations of the natural orbitals, which are obtained by diagonalizing the subspace density matrices ρ̂^(κ)^(*t*).^55^ If the highest natural orbital is nearly unoccupied, addition of further SPFs will have a negligible effect, showing that convergence has been reached.

As in the case of MPS wave functions, the propagation error is mainly due to the projection error, i.e., here an insufficient number of SPFs. Adaptive procedures have been developed in order to minimize the local-in-time error,^81,82^ which quantifies the projection error during the propagation. Furthermore, systematic strategies to optimize the multilayer tree structure have been proposed such as to reduce the propagation error.^49^

## 3 Results

### 3.1 Model A

**Figure 4:**
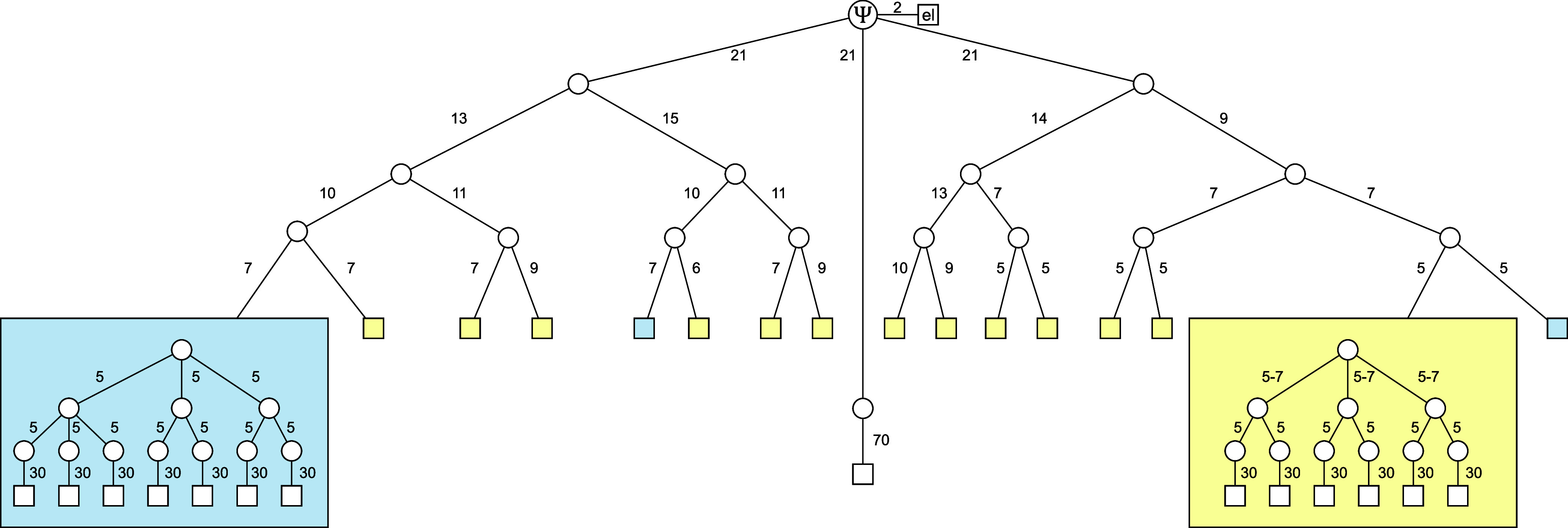
Multilayer tree of the ML-MCTDH wave function for model A with up to 6 layers. The wave function is partitioned into an electronic branch (el), two branches for the tuning modes (left-hand side and right-hand side), and one branch for the intermolecular mode (center). Parts of the tree that are equivalent by symmetry are highlighted by boxes of the same color. Circles represent nodes and rectangles represent the primitive basis of the respective phonon modes. Numbers next to lines connecting two nodes indicate the number of SPFs, and numbers next to lines connecting nodes and phonon modes indicate the number of primitive basis functions. For symmetry equivalent sub-branches that are not explicitly shown, the range of SPFs is indicated with the smallest and highest number of SPFs.

We carefully perform convergence checks of the numerical parameters of the MPS and ML-MCTDH calculations. Their results are summarized in Section S4 of the Supporting Information. On the MPS side, these include the maximally allowed bond dimension during the time evolution and the maximum number of oscillator states taken into account. For the ML-MCTDH simulations, the number of SPFs is adjusted across the multilayer tree. The multilayer tree used in the ML-MCTDH calculation is shown in Figure 4.

For the MPS calculation, we arrange the vibrational factor spaces such that the R-mode is right next to the electronic factor space. The remaining vibrational factor spaces are arranged next to one another ordered with decreasing mode frequency. These convergence tests indicate that both methods are close to convergence. For example, focusing on the occupation dynamics as observable, we find for MPS calculations employing the numerical convergence parameters *N*_b_^+^ = 14 and *N*_b_^+^ = 18 that the maximum difference is below 10^–3^ throughout the entire simulation time of 200 fs (cf. Figure S2(a) in the Supporting Information). Similarly, comparing the same quantity in the ML-MCTDH approach for 18 and 21 SPFs in the top layer, we find deviations of comparable size in Figure S3(a). A convergence check of the natural orbital populations,^55^ as usually done for ML-MCTDH calculations, shows that highest values of around 5 × 10^–4^ (both in the upper layer as well as when taking all layers into account) are found.

#### 3.1.1 Description of the Electronic State Occupancy

**Figure 5:**
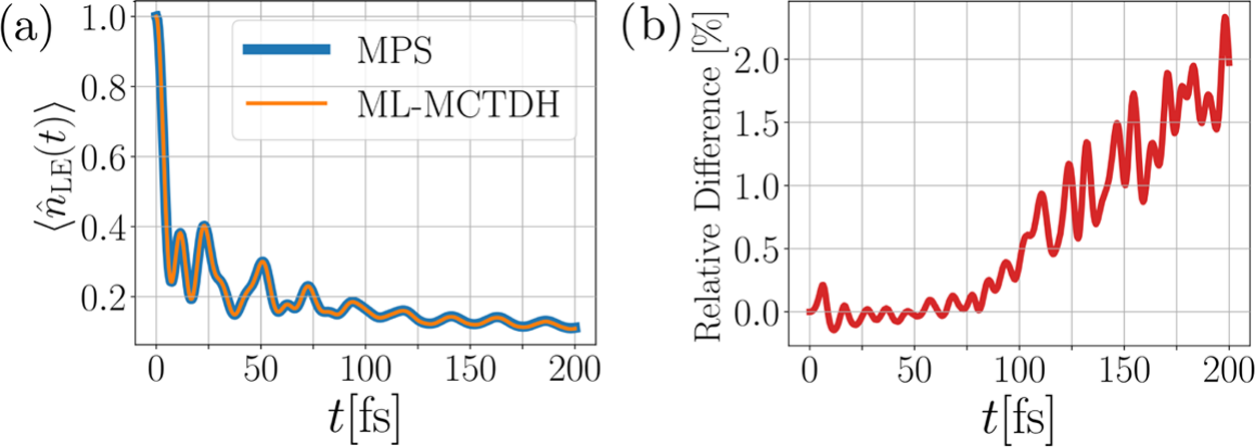
Comparison of the MPS and ML-MCTDH method for the LE state occupation over time for model A. We find that both methods give highly consistent results. Panel (a) displays the evolution of the LE state occupation for both methods over time, while panel (b) depicts the relative difference between the MPS and ML-MCTDH result.

Let us now come to an actual comparison between the MPS and ML-MCTDH methods by first investigating the dynamics of the LE state occupancy over time. Although the off-diagonal electronic coherences would also be interesting quantities,^43^ we focus here on the state occupancies. We plot the computed state occupancies ⟨*n̂*_LE_(*t*)⟩ from both methods in Figure 5(a), showing the depopulation of the LE state into the CT state. The time scale of τ ≅ 50 fs indicates ultrafast charge separation at the molecular interface. On the considered time scale, the agreement between the two methods is excellent. Deviations can only be found at the very end of the dynamics, but are barely visible on the scale of Figure 5(a). Plotting the relative difference between the MPS and ML-MCTDH results in Figure 5(b) unveils the magnitude of the deviation. While up to about 75 fs, the relative difference, which we define equally throughout this paper as
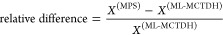
15with X = ⟨*n̂*_LE_⟩ in this case, oscillates around zero with small amplitude, it starts to increase beyond this time. Despite these differences, the deviations remain orders of magnitude smaller than the actual occupancy up to *t* = 200 fs, such that in this particular case one can conclude that both methods treat the state occupation essentially identically.

#### 3.1.2 Description of Bath and Intermolecular Modes

**Figure 6:**
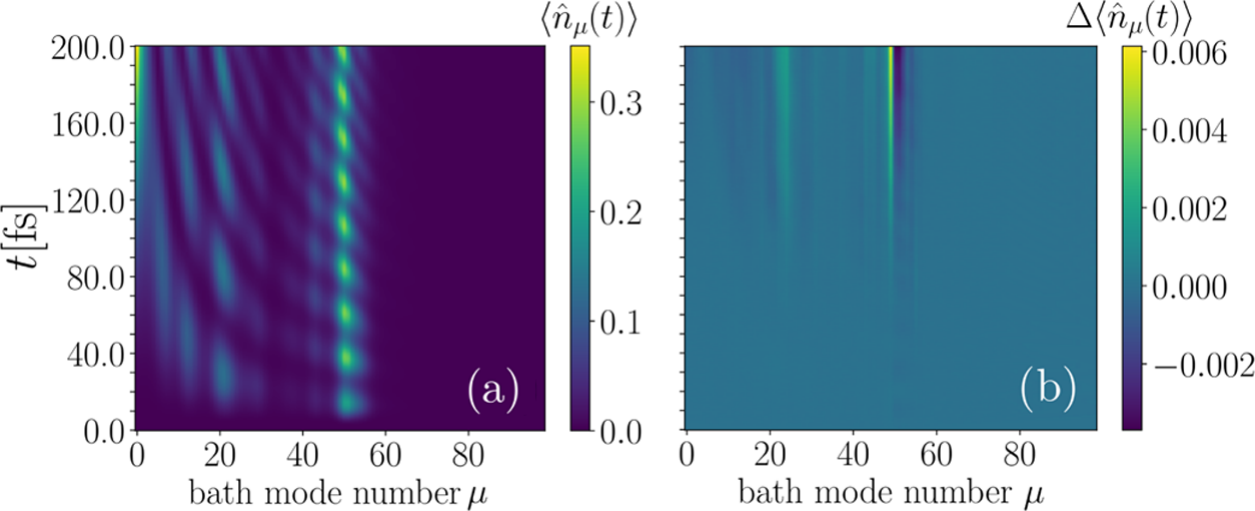
Comparison of the MPS and ML-MCTDH method for the bath mode occupations over time for model A. We find that MPS and ML-MCTDH describe the bath in an equivalent way. Panel (a) displays the evolution of the occupation number computed via MPS approach over time. Panel (b) shows the difference between the MPS and ML-MCTDH result.

To investigate the differences of the MPS and ML-MCTDH calculations more deeply, we further consider the occupation number of the modes over time. Let us first focus on the bath modes. In Figure 6(a), we display the occupation number dynamics of all of the bath modes computed via the MPS approach. Here, we observe mainly the typical polaronic oscillations in the occupation of the more strongly coupled modes as a sign of their involvement in the dynamics of the system.

For the comparison of the MPS and ML-MCTDH approach for this quantity, we plot the difference of the occupation numbers over time in panel (b). We observe that minor deviations start to appear around 75 fs. In the considered case, there is no systematic trend concerning the sign of deviation of the MPS from the ML-MCTDH result, as the different color coding for the difference shows. Still, the deviations are, as in the case of the state occupations, orders of magnitude smaller than the actual value of the occupation, such that we again conclude that the MPS and ML-MCTDH results can be considered equivalent.

**Figure 7:**
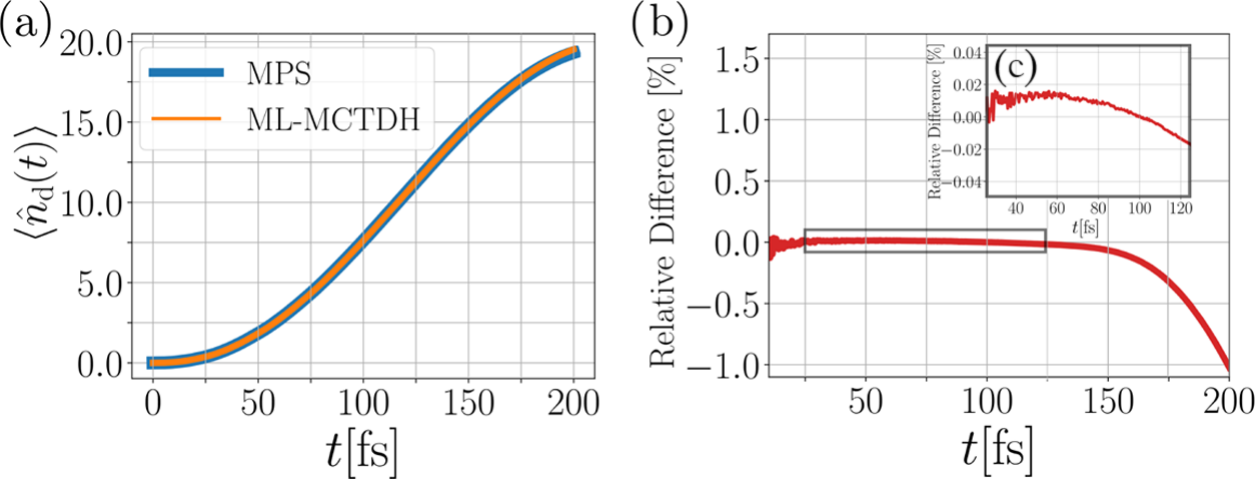
Comparison of the MPS and ML-MCTDH method for the intermolecular mode occupations over time for model A. As depicted in panel (a), we find for the dynamics of the intermolecular mode occupation, similar to the state occupations, only minor differences. While panel (b) indicates that the deviation close to the end of the considered time scale, around 150 fs, are possibly influenced by the different basis representation in the two methods, the deviation start earlier at around 75 fs as visible in panel (c), similar to the state occupations.

As a last part of this subsection, we focus on the description of the dynamics of the occupation number of the intermolecular mode. This mode modifies the dynamics in a non-negligible way, even though the time scale of charge separation remains unaffected.^42,43^ We compare the phonon occupation of this mode over time computed with both approaches in Figure 7. Analyzing the dynamics of its occupation, we observe only slight variations between MPS and ML-MCTDH, akin to those found in the state occupation (cf. Figure 7(a)). Deviations toward the end of the examined evolution time, i.e., at approximately 150 fs, are evident in Figure 7(b) and could be affected by the fixed harmonic oscillator basis in the MPS calculation. Notably, these deviations start to appear earlier, around 75 fs, aligning with observations in the state occupancy. The origin of these deviations will be considered later on.

#### 3.1.3 Entanglement Entropy between the Electronic and Vibrational System

While up to now we studied observables that either involve only the electronic system or the vibrational subsystem, a relevant quantity that characterizes the interaction of the two subsystems is the entanglement entropy between both and its evolution over time. This quantity can be defined as
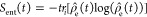
16where ρ̂_e_(*t*) = tr_λ_[ρ̂(*t*)] denotes the reduced density operator for the electronic subsystem and ρ̂(*t*) is the full time-dependent density operator of the system. Our examination reveals intriguing temporal patterns in the entanglement dynamics. Initially, within the first few femtoseconds, we observe a pronounced entanglement rise, peaking below the maximally possible value of ln(2) ≈ 0.693. This indicates the built-up of robust entanglement of the electronic subsystem with the phonon system. This is dominated by specific modes on that time scale, notably within the 180–200 meV range. Subsequently, the entanglement entropy reduces as the system localizes in the charge-transfer (CS) state. This time scale can be characterized by the emergence of a close-to-static polaron state, characterized by a dressing of the CS state. This is accompanied by discernible fluctuations in population attributed to the intermolecular mode and the transfer integral. The formation of this quasi-static polaron state manifests not only in the electronic degrees of freedom but also within the vibrational bath, evidenced by observable polaronic oscillations within the set of relevant modes in Figure 6. This evolution leads to a significant decrease in entanglement between the electronic and vibrational subsystems.

**Figure 8:**
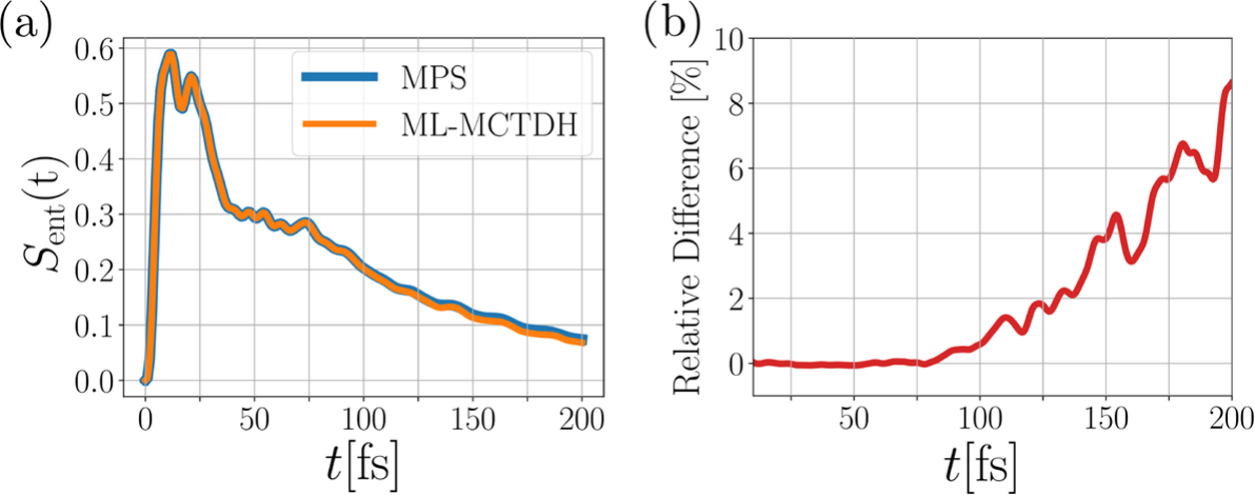
(a) Comparison of MPS and ML-MCTDH methods for the entanglement entropy over time for model A. Panel (b) shows the relative difference of the entanglement entropy between the MPS and MCTDH approaches. Similar to the other quantities considered previously, we find good agreement between the two methods for the description of the entanglement between the electronic and vibrational subsystem. However, deviations are larger than before, ranging up to 8% until the simulation time maximum.

By comparing the two methods of interest regarding the computation of the entanglement entropy of the electronic and vibrational system, we find in Figure 8(a) that both methods in general agree very well over the full simulation time. However, as in the previously considered observables, differences start to appear at around 75 fs and increase up to 8% until the end of the simulation. It is evident that the MPS approach predicts a slightly stronger entanglement between the electronic and vibrational system on longer time scales.

#### 3.1.4 Discussion and Possible Origins of Deviation

Let us summarize the findings of this section. We have compared the dynamics of the state occupancy of the LE state, the occupation of the different phonon numbers, and the entanglement entropy of the electron and vibrational subsystems. In all of the cases, we found very good agreement between the MPS and the ML-MCTDH approaches. We identified that minor differences consistently start to appear on a single time scale, which we found to be at around 75 fs. Although both methods employ the harmonic oscillator basis in the bottom layer, their individual representation is slightly different. While the ML-MCTDH uses a harmonic oscillator DVR with *N*_DVR_ = 30 basis function, the MPS approach uses the conventional harmonic oscillator eigenstate basis and their analytic matrix elements.

**Figure 9:**
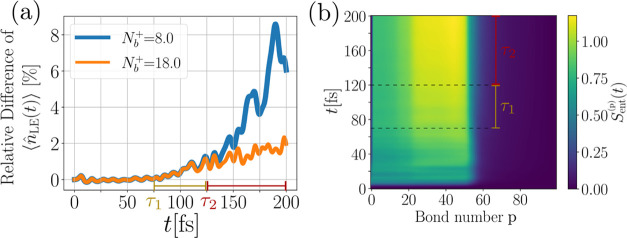
(a) Comparison of the relative deviation of the electronic LE state occupation of the MPS approach for two different basis sizes to the ML-MCTDH method. This shows that the time scale of deviation τ_1_ = 75 fs is not related to the slightly different basis used in the MPS implementation. Panel (b) shows the bipartite entanglement entropy as a measure of the entanglement of the modes over time computed with the MPS approach. We find on exactly the time scale τ_1_ an increase in the entanglement entropy of the subsystems  up to  (see main text). This indicates that the deviation between ML-MCTDH and MPS is related to the nonidentical distribution of entanglement between the individual degrees of freedom, as a result of the different network structure.

To exclude that the observed differences are related to the different (time-independent) basis implementation and hence possibly different convergence properties, we compare the relative difference of the ML-MCTDH and MPS results in Figure 9(a). The MPS data has been simulated for two different *N*_b_^+^ values. If the deviations between MPS and ML-MCTDH were related to the different basis representation, one would expect that the particular time at which the deviation between the two methods occur, will depend on the chosen *N*_b_^+^. However, as visible in Figure 9(a), this is not the case. The figure shows that the time τ_1_ where the deviation to the ML-MCTDH method starts rising is independent of the basis (indicated as a time window in Figure 9(a)). Therefore, the original source of deviation is not related to the basis in the MPS implementation. In contrast, at a later time τ_2_, the dynamics of the system described by the smaller basis deviates in an exponential fashion from the ML-MCTDH result, which is then indeed related to the smaller basis for *N*_b_^+^ = 8.

Thus, there must be another reason for the deviation between the two methods. While in principle both methods can recover the exact quantum dynamics in a certain limiting case, this limit is practically never reached, due to the exponentially growing numerical cost. As a consequence, both methods are intrinsically approximations to the exact solution. This means that deviations visible around τ_1_ = 75 fs may be just related to the different tensor network structure used and to how this structure allows the flow of correlation and entanglement between the subsystems for a finite dimensional approximation used in practice.

One indication for this hypothesis can be found by examining the bipartite entanglement entropy *S*_ent_^(*p*)^(*t*) from the MPS calculation. In the MPS calculation, the local Hilbert spaces of the modes are ordered with increasing mode energy, with the electronic degrees of freedom at the left end of the tensor network. Thus, the full Hilbert space  decomposes as
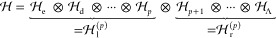
17into a left  and right part  with respect to bond index *p*. With this ordering fixed, the bipartite entanglement entropy can be defined as
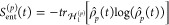
18where  is the reduced density matrix of subsystem . It encodes how strongly the left system is entangled with the right system, and can therefore be used to study how the different vibrational modes of our model are entangled among each other, mediated by the electronic system, and how this entanglement evolves during the dynamics. In Figure 9(b), we plot this quantity as a function of the bond number *p* over time. We observe a significant increase in the entanglement entropy of the subsystems *p* = 25 (related to vibrational mode ω_25_ = 91.08 meV) which starts at around τ_1_. Strong entanglement persists up to *p* = 50 (ω_50_ = 182.16 meV). Beyond this index, the bipartite entanglement drops substantially. This entanglement is probably related to two-phonon up or down conversion processes, due to the closely matching energy of such processes for the involved modes. Regardless of the individual processes leading to this entanglement, the time scale of this increase of entanglement aligns with the deviation time scale τ_1_. This supports the hypothesis that the deviation between the two methods is related to the intrinsically different ways entanglement can flow through the system, as dictated by the structure of the tensor network.

### 3.2 Model B

While the above model A only admits a relatively limited entanglement entropy between the electronic and vibrational system, captured by both methods in a proper way, model B features a larger number of electronic states, and is therefore potentially more entangled. Building on the above findings on the smaller model, model B is therefore a more complex test case. Before we start the comparison between the two methods, let us summarize the results of the convergence tests, which can be found at full length in the Supporting Information (Section S5). These tests confirm the MPS approach’s validity with a maximum bond dimension of 400 and *N*_b_^+^ = 18. The potentially stronger entanglement in model B is indeed confirmed by the ultrafast increase in the maximum bond dimension of the MPS reaching *D* = 400 for a cutoff for the Schmidt values of 10^–13^ in less than 20 fs. Complementary to these tests of the MPS approach, we tested the convergence of the ML-MCTDH method. The results indicate that we have satisfactorily converged simulation with up to 40 SPFs in the top layer and varying numbers of SPFs in the lower layers (cf. Figure S5 of the Supporting Information where convergence in terms of natural orbital populations^55^ around 2 × 10^–3^ is demonstrated).

**Figure 10:**
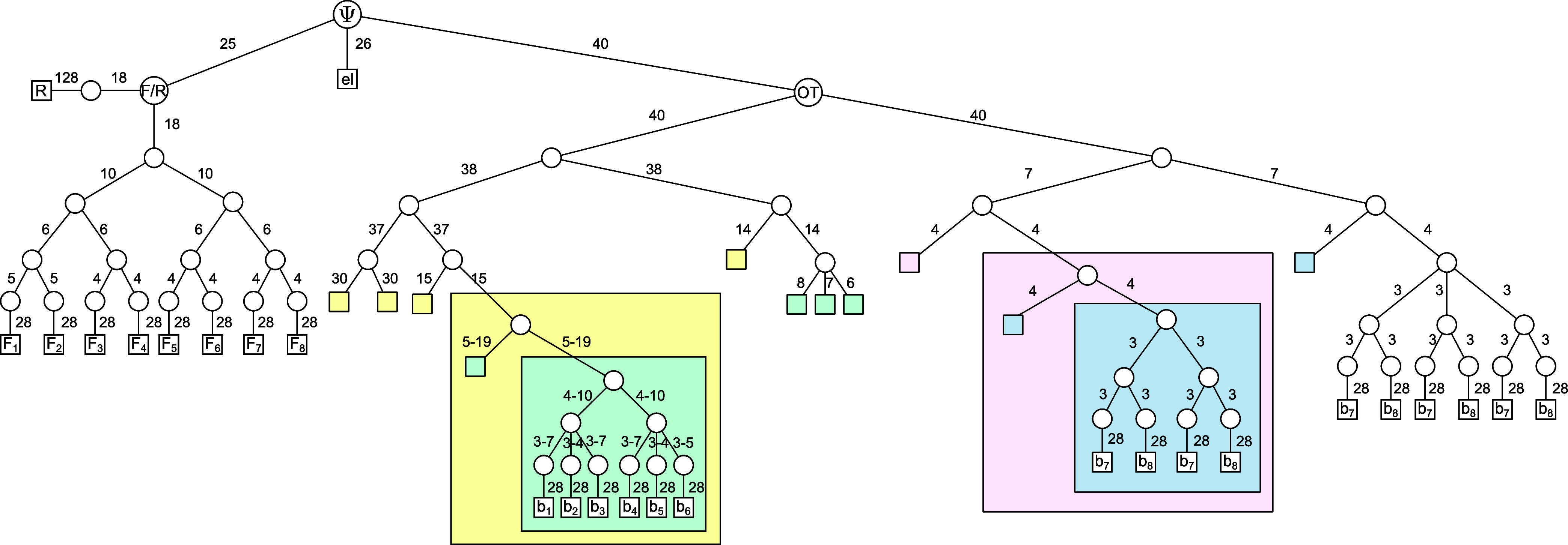
Multilayer tree of the ML-MCTDH wave function for model B with up to 8 layers. The wave function is partitioned based on the physical system into an electronic branch (el), a branch for the fullerene modes and intermolecular mode (F/R), and a branch for the thiophene modes (OT). The thiophene branch is further divided into a sub-branch for the low- and mid-frequency modes including CC stretch modes (up to 1500 cm^–1^) and a sub-branch for the highest-frequency modes of CH type (above 3000 cm^–1^). The meaning of boxes, circles, and rectangles as well as specifying numbers is equivalent to Figure 4.

The multilayer tree underlying the ML-MCTDH calculations is shown in Figure 10 and comprises up to *Q* = 8 layers. As in model A, the electronic degrees of freedom are collected into a particle at the top of the tree, in line with the so-called single-set approach. The phonon part of the tree is then structured such as to mimic the fragment-based nature of the Hamiltonian, which determines the vibronic connectivities, noting that most vibrational modes are local modes (with the exception of the *R*-mode). Following earlier work, effective phonon modes for the acceptor (fullerene) and donor (oligothiophene) moieties were obtained from first-principles-computed spectral densities for the different types of electronic states.^46,83^ The phonon space is divided into a first subspace comprising the interfacial distance mode and the fullerene modes that are exclusively coupled to the charge-separated states, and a second subspace for the oligothiophene modes that couple to both Frenkel exciton and charge-separated states. The second, oligothiophene, sub-branch is further divided into separate branches for modes up to 1500 cm^–1^, and the remaining modes over 3000 cm^–1^. These branches are finally divided according to the system’s fragment structure, as can be seen in the penultimate layer of the tree. The final layer represents the primitive basis. For comparison, we constructed an alternative tree, shown in the Supporting Information (Figure S9), that divides the oligothiophene sub-branch solely based on the fragment structure. Both trees give almost the same results.

In the MPS calculation, we place the *R*-factor space directly adjacent to the electronic factor space. The other modes are arranged blockwise. The first block of factor spaces, next to the *R*-mode factor space, is associated with the modes of the fullerene super particle, arranged in order of decreasing frequency. The next block corresponds to the modes of the OT fragment at the interface, also arranged in the order of decreasing frequency. This procedure is repeated for the other OT fragments. To get an estimate of how strongly the result is affected by a different ordering, we compare the cases where the *R*-mode is placed at the beginning/end of the chain. We find negligible differences.

#### 3.2.1 Description of the Electronic State Occupancy

**Figure 11:**
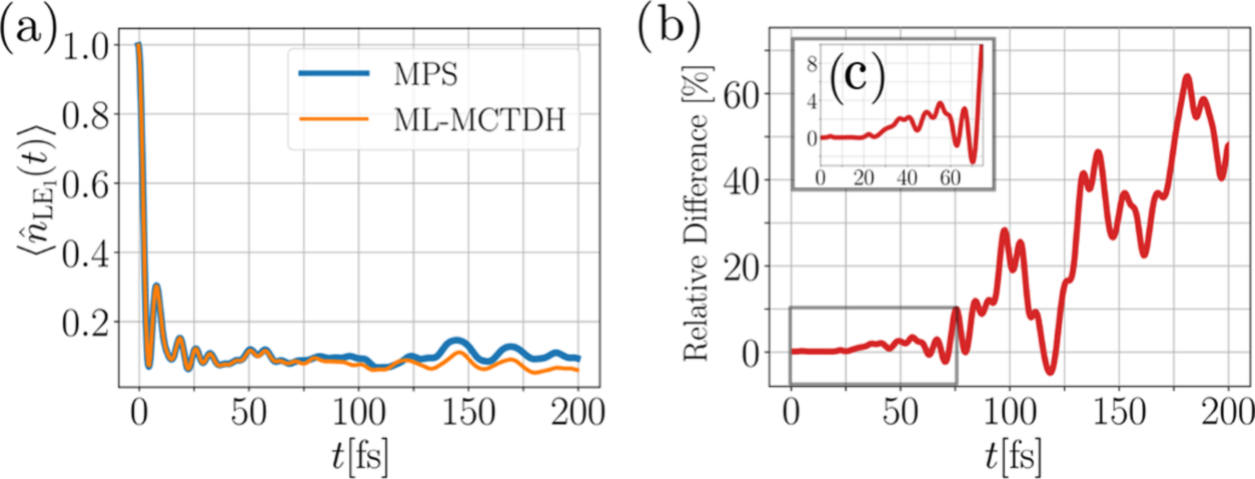
Comparison of the MPS and ML-MCTDH method for the occupancy of the LE_1_ state for model B.

We now compare the nonadiabatic electronic dynamics computed with the ML-MCTDH and MPS approaches, and illustrate the state population of the LE_1_ state over time. Here, we focus exclusively on the population dynamics. We expect a similar comparison for the off-diagonal coherences, but have not studied these explicitly. We plot the results of this comparison in Figure 11. There we find that both methods give nearly indistinguishable values up to a time scale of 20 fs and then start to deviate (cf. Figure 11(c)). This behavior is similar to the one of model A, but the deviations here are much more significant, reaching a relative difference of the two methods of up to 60% until the end of the simulation time. Still, as visible in Figure 11(a), the dynamics is rather close up to 60 fs and even on a later time scale, the oscillation features remain qualitatively the same.

**Figure 12:**
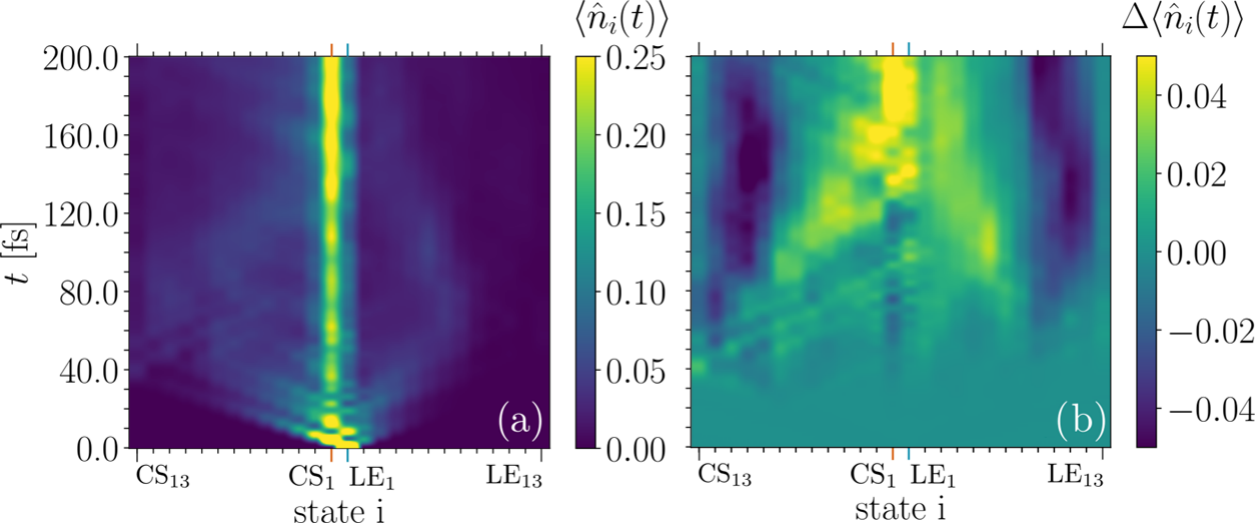
(a) Electronic state occupations over time computed via the MPS approach. (b) Difference of the state occupancy as a function of time computed with the MPS and ML-MCTDH methods. At short time scales, both methods describe the dynamics equivalently. On later time scales, the MPS approach localizes the charge more strongly in the proximity of the CS_1_ state (yellow), while within ML-MCTDH, the density is more strongly delocalized (blue).

In addition to the occupancy of the LE_1_ state, we extend our study and consider the electronic dynamics of all 26 electronic degrees of freedom more globally. In Figure 12(a), we plot the state populations computed via the MPS approach over time. At the earliest times, the initial charge transfer from the LE_1_ state to the CS_1_ state starts the dynamics. Subsequently, partial charge separation occurs, followed by the emergence of long-range CS states and partial trapping of the exciton in the vicinity of the CS_1_ state. For the comparison between the two methods, we plot the difference in the populations over time in Figure 12(b). In addition, Supporting Information Section S6 compares the time-evolving state occupancies for the individual CS and LE states. In Figure 12(b), one first observes that it takes a certain time until deviations reach a certain site away from the initial site. For the long-range separated CS states, it takes up to 40–50 fs until deviations between state occupations become visible. We also observe traces of the backscattering at the boundary of the system at around 60 fs, which seem to be described in a similar fashion by both methods. On the other hand, there are also pronounced differences. The states that are more distant from the initial one feature higher population in ML-MCTDH, while the MPS approach seems to localize the state occupancy closer to the first OT fragment. This characteristic is enhanced over time. The radius of localization around the OT_1_ fragment in the MPS dynamics shrinks over time, but the CS_*n*_, *n* = 1, ..., 4, states always remain more strongly populated than predicted by ML-MCTDH (cf. Supporting Information Section S6). Conversely, the long-range charge-separated states (CS_*n*_, *n* = 7, ..., 13) are significantly less populated in the MPS calculation as compared with the ML-MCTDH result, to the point that the deviation between the mean of the integrated free carrier populations *n̂*_free_=∑_*l*=7_^13^*n̂*_CS,*l*_ reaches nearly 50% (cf. Supporting Information Figure S7).

In summary, we find that in this case, the ML-MCTDH approach leads to a stronger delocalization of the wavepacket, while the MPS method localizes the state population increasingly strongly close to the CS_1_ state.

#### 3.2.2 Entanglement Entropy between the Electronic and Vibrational Subsystems

**Figure 13:**
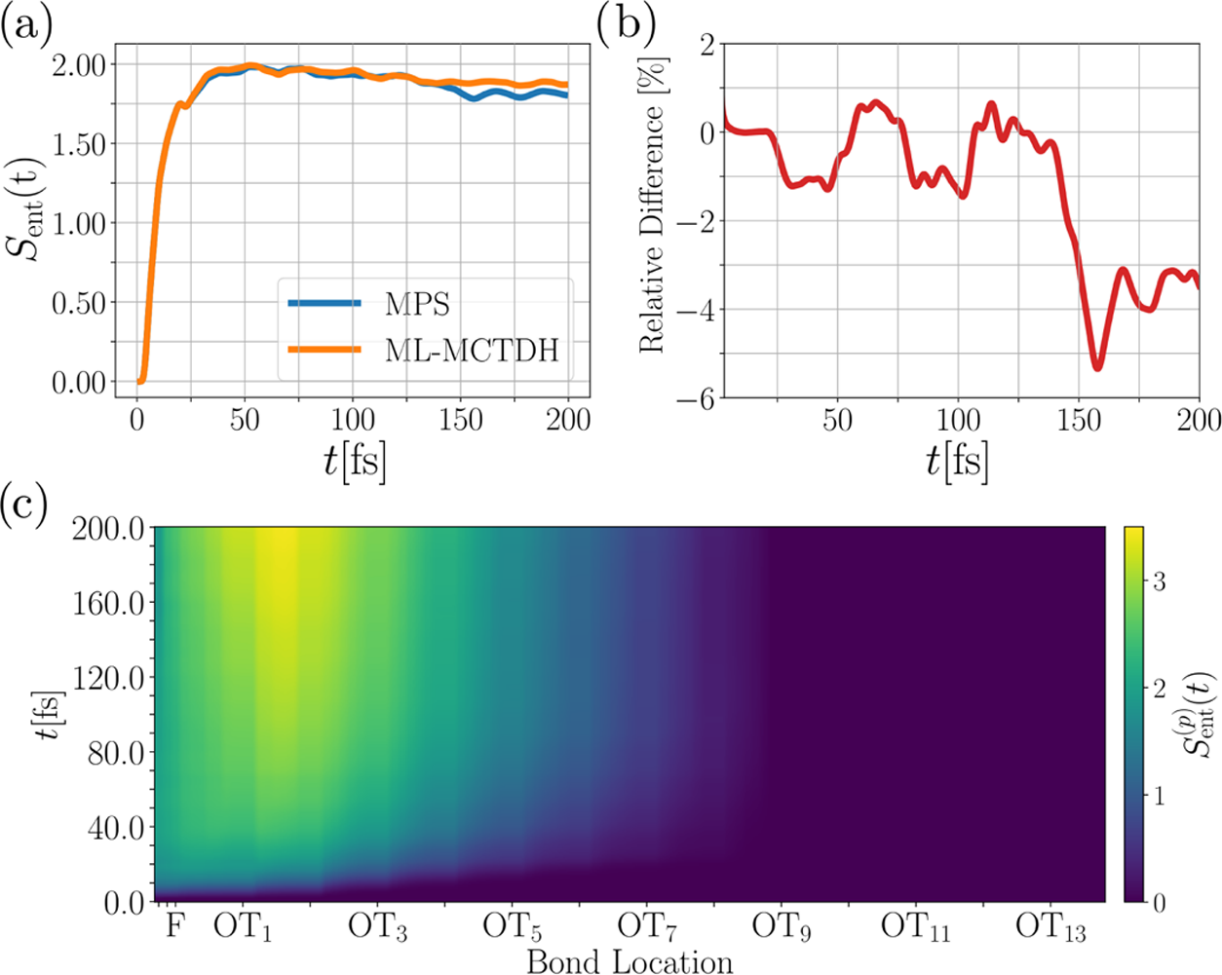
(a) Comparison of the entanglement entropy between the electronic and vibrational subsystems between the MPS and ML-MCTDH method over time. (b) Relative difference of the curves in panel (a). (c) Bipartite entanglement entropy by cutting the state at bond (*p*) over time computed with the MPS method.

As the entanglement entropy *S*_ent_(*t*) between the electronic and vibrational subsystems (eq 16) proved useful as an indicator for the difference between the ML-MCTDH and the MPS dynamics in model A, we shall now also focus on this quantity. We compare *S*_ent_(*t*) computed by the two methods in Figure 13(a) depicting the overall behavior. Here, one can observe an initial, and very intense, increase in the entanglement entropy, which is described by both methods. A small kink is visible in the entanglement entropy around 25 fs, which is described identically by both methods. After that, both methods start to deviate within ∼1% but both methods give a similar curve shape. One method sometimes yields a higher entanglement entropy than the other, and vice versa. The roles of the methods in producing higher entropy appear to oscillate up to about 125 fs.

Beyond this time, the entanglement entropy in the MPS approach starts to fall below the ML-MCTDH value, which we attribute to localization behavior. Localization, as seen more pronounced in the electronic dynamics of the MPS approach, can give rise to a less entangled vibrational and electronic system. The plot of the bipartite entanglement entropy, shown in Figure 13(c), reveals that the modes of the OT molecule at the interface are especially entangled in this localization process in the MPS dynamics. Recalling the time of about 60 fs upon which the free carrier populations in the MPS and ML-MCTDH calculations start deviating, we can identify this with the time of maximum entanglement between the electronic and vibrational subsystems. After this time, the initial entanglement surge gives way to the relatively steady increase of bipartite entanglement (cf. Figure 13(c)), indicating the involvement of the vibrational modes around the interface. Although the bipartite entanglement entropy is not directly accessible in ML-MCTDH, the match of these times seems to indicate, similar to model A, that it is the different description of entanglement between the subsystems that is causal for the deviations between the methods. However, compared to model A, the deviation between the methods is much more pronounced, probably as a result of the multi–site character and overall stronger entanglement in model B.

## 4 Discussion

To summarize our findings, the MPS and ML-MCTDH results align very well with only minor differences for model A, but this is far less the case for model B: Here, we found after about 60 fs significant quantitative differences in the description of the observables under study, while the qualitative physical picture remains the same. Since both types of calculations can be considered satisfactorily converged, the reason for these differences likely lies in the different types of tensor networks underlying the MPS vs ML-MCTDH approaches, guiding the entanglement flow between the individual degrees of freedom. Specifically, we found that the MPS approach leads to a stronger localization of the dissociated exciton in the vicinity of the CS_1_ state, involving non-negligible occupancies of CS_*n*_, *n* = 2, ..., 4, while the free carrier populations (CS_*n*_, *n* = 7, ..., 13) are less populated in the MPS calculations. The latter can be quantified by an integrated free carrier populations that is reduced by up to 50% as compared with ML-MCTDH.

For both models studied, we could identify that prior to deviations in electronic population and mode occupation, differences in the entanglement entropy *S*_ent_ could be observed. While for the first model, *S*_ent_^(MPS)^ is at all times larger than *S*_ent_^(MCTDH)^, this quantity shows a oscillatory behavior in model B. That is, up to *t* ≤ 125 fs, *S*_ent_^(MPS)^ and *S*_ent_^(MCTDH)^ are very similar apart from slight oscillations, within a range of about 1%. On longer time scales, the entanglement entropy in the MPS calculation decreases more strongly than in the ML-MCTDH calculation. This is consistent with the different dynamical evolution of the state populations, as around *t* ≅ 150 fs, the exciton is more strongly localized around the OT_1_ fragment in the MPS approach than in the ML-MCTDH method. This leads to a slightly larger entropy for the ML-MCTDH calculations at longer times. Still, the entanglement entropy between the electronic and vibrational subsystem remains rather close. Another interesting aspect is the match between the deviations of the bipartite entanglement entropy and the time where deviations in the population, especially the free carrier population, appear. These observations hint toward a different treatment of the entanglement between the individual degrees of freedom, which may be related to the different tensor network structure of the two methods and consequently different convergence properties.

In the literature, there are conjectures regarding the enhanced capability of tree tensor networks, such as ML-MCTDH, compared to MPS, in capturing long-range correlations in critical systems or for systems with long-range interactions.^59^ As this also includes the electron–phonon interaction, these statements are highly relevant for the two models under study in this article. However, at least for model A, this does not appear to be the case. The entanglement entropy, as a measure of correlation, is here consistently larger in the MPS simulation compared to ML-MCTDH.

Because of the mentioned small oscillations in the entanglement entropy, the situation is more intricate in model B. Here, we encounter the interesting situation that the entanglement entropy, as a measure for the correlation, is similarly described in the MPS and ML-MCTDH calculations on an intermediate time scale, but longer-range spatial correlations seem to be favored by ML-MCTDH (see the above discussion). On the other hand, one can also not finally conclude about the converse statement, as there are many other factors of difference beyond the network topology. This includes the employed time-evolution algorithm, the different arrangement of the individual factor spaces, partitions of the tree in either tensor network, or the model under consideration, which may have an impact. In the case of model B, we compared with an alternative tree structure, featuring a modified sub-branch ordering of low vs high- frequency phonon modes. The differences in electronic occupancies remained minor, though.

Clearly, it would be highly desirable to have exact benchmark calculations for the present system. In absence of these, it remains unclear at this point, and possibly also in the nearest future, whether the MPS or ML-MCTDH calculations are closer to the exact dynamics, in particular regarding the aspect of long-range charge separation. Also, while both methods are regarded as quasi-exact in their respective field, this status has to be re-examined in the light of the present results for these large systems. We would like to emphasize that both cannot account for an exponentially growing Hilbert space and therefore rely on individual approximations. While some intrinsic limitations of MPS have been partially addressed within methods of quantum information theory, in terms of the respective area laws^19,84^ for quantum lattice models, a comprehensive study of limitations of different network structures in a more general context is still missing.

In conclusion, despite the quantitative differences that we unveiled, our results demonstrate that both methods effectively capture the same qualitative physics and both give a handle on describing the transition between coherent and relaxation phenomena in large systems. In any event, both approaches are far better suited to describe the nonadiabatic dynamics in large systems than many semiclassical or quantum-classical schemes.

## Supporting Information Available

Benchmark simulations; a detailed comparison of state populations; specific model Hamiltonians and respective parameters as well as an alternative ML-MCTDH tree for model B (PDF)

## Footnotes

1 Link to the TDVP code: https://github.com/ITensor/TDVP (accessed 12 May 2021)
