# Supplementary material for: Comparison of Matrix Product State and Multiconfiguration Time-Dependent Hartree Methods for Nonadiabatic Dynamics of Exciton Dissociation
Source: J Chem Theory Comput. 2024 Oct 4;20(20):8767–81. doi: 10.1021/acs.jctc.4c00751 (PMC11500411; doi:10.1021/acs.jctc.4c00751)
Supplement: Supplementary file 1 — ct4c00751_si_001.pdf [file ct4c00751_si_001.pdf]

Supporting Information: Comparison of Matrix-Product-State and Multiconfiguration Time-Dependent Hartree Methods for Non-Adiabatic Dynamics of Exciton Dissociation

Maximilian F. X. Dorfner[1], Dominik Brey[2], Irene Burghardt[2],* and Frank Ortmann[1*]

[1] *TUM School of Natural Sciences, Technische Universität München, 85748 Garching b. München, Germany*

[2] *Institut für Physikalische und Theoretische Chemie, Goethe Universität Frankfurt, 60438 Frankfurt am Main, Germany*

* E-mail: burghardt@chemie.uni-frankfurt.de

* E-mail frank.ortmann@tum.de

S1 Benchmark for the MPS Time-Evolution Protocol

To provide further confidence in the validity of the MPS results presented in the main text, we benchmark here the time-evolution protocol. The first property is the norm conservation. When considering Model B with a maximum bond dimension of 250 we find maximum deviations up to 3×10^{-6} over the full time range of 200 fs.

To provide a further independent benchmark, we consider the Hamiltonian

$$\hat{H} = \sum_{i=1}^{9} t \left[|i\rangle \langle i+1| + |i+1\rangle \langle i| \right] + \sum_{i=1}^{10} g_i \, |i\rangle \langle i| \left[\hat{b}_i + \hat{b}_i^\dagger \right] + \sum_{i=1}^{10} \omega_i \hat{b}_i^\dagger \hat{b}_i, \tag{S1}$$

with 10 electronic states and 10 linearly coupled phonon modes. We choose $t = 100$ meV, $g_i = 100$ meV and $\omega_i = 150$ meV. We compute the time-evolution of the initial state $|1\rangle$ with respect to this Hamiltonian for the first 100 fs, by either the MPS time-evolution protocol presented in the main text and alternatively by employing a global Runge-Kutta method (fourth order, timestep of 0.25 fs). We take into account 12 oscillator states per mode and limit the maximum bond dimension to 300. We plot the population dynamics of state $|10\rangle$ in Fig. S1. As visible there, we have perfect agreement between the two methods. This supports the validity of the time-evolution scheme used in the main text.

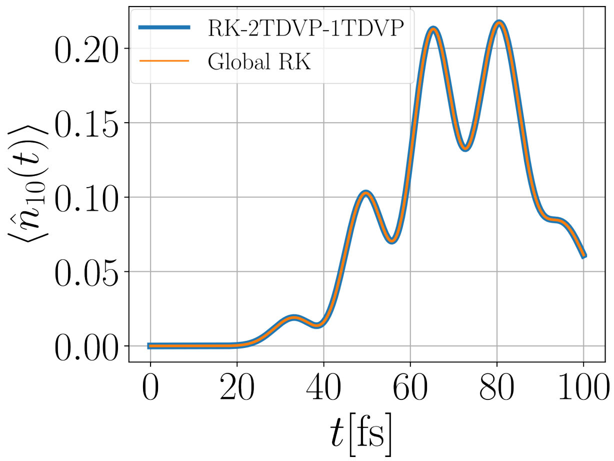

Figure S1: Population dynamics of state 10 arising from initial state $|1\rangle$ for the Hamiltonian Eq. (S1) computed with either a global Runge-Kutta method or the protocol presented in the main text. We find perfect agreement between both approaches.

S2 Hamiltonian of Model A

The explicit form of the Hamiltonian underlying model A is given by

$$\begin{aligned}\hat{H} &= \epsilon_{\mathrm{CS}} \left|\mathrm{CS}\right\rangle \left\langle\mathrm{CS}\right| + t_{\mathrm{LE,CS}} \left[\left|\mathrm{CS}\right\rangle \left\langle\mathrm{LE}\right| + \left|\mathrm{LE}\right\rangle \left\langle\mathrm{CS}\right|\right] \\ &+ \sum_{\mu} g^{\mu}_{\mathrm{CS,CS}} \left|\mathrm{CS}\right\rangle \left\langle\mathrm{CS}\right| \left[\hat{b}^{\dagger}_{\mu} + \hat{b}_{\mu}\right] + g^{R}_{\mathrm{CS,CS}} \left|\mathrm{CS}\right\rangle \left\langle\mathrm{CS}\right| \left[\hat{b}^{\dagger}_{R} + \hat{b}_{R}\right] \\ &+ g^{R}_{\mathrm{LE,CS}} \left[\left|\mathrm{CS}\right\rangle \left\langle\mathrm{LE}\right| + \left|\mathrm{LE}\right\rangle \left\langle\mathrm{CS}\right|\right] \left[\hat{b}^{\dagger}_{R} + \hat{b}_{R}\right] + \omega_R \hat{b}^{\dagger}_{R} \hat{b}_{R} + \sum_{\mu} \omega_{\mu} \hat{b}^{\dagger}_{\mu} \hat{b}_{\mu}. \end{aligned} \tag{S2}$$

In this study we used the values $\epsilon_{\mathrm{CS}} = -79.0$ meV, $t_{\mathrm{LE,CS}} = 130.0$ meV, $g^{R}_{\mathrm{CS,CS}} = 30/\sqrt{2}$ meV and $g^{R}_{\mathrm{LE,CS}} = -10/\sqrt{2}$ meV. The respective parameters for the tuning modes ω_{μ} and $g^{\mu}_{\mathrm{CS,CS}}$ can be found in Tab. S1.

Table S1: Mode frequencies and coupling strengths for model A. All quantities in meV.

μ	ω_μ	$g^\mu_{\mathrm{CS,CS}}$	μ	ω_μ	$g^\mu_{\mathrm{CS,CS}}$	μ	ω_μ	$g^\mu_{\mathrm{CS,CS}}$	μ	ω_μ	$g^\mu_{\mathrm{CS,CS}}$
1	3.643	2.511	26	94.722	12.158	51	185.800	47.272	76	276.879	2.822
2	7.286	2.359	27	98.365	11.779	52	189.443	47.873	77	280.522	2.709
3	10.929	2.347	28	102.008	12.196	53	193.087	43.415	78	284.165	2.605
4	14.573	2.586	29	105.651	13.061	54	196.730	39.088	79	287.808	2.509
5	18.216	3.190	30	109.294	13.549	55	200.373	34.622	80	291.451	2.420
6	21.859	4.203	31	112.937	12.606	56	204.016	28.686	81	295.095	2.338
7	25.502	5.224	32	116.581	10.303	57	207.659	22.148	82	298.738	2.262
8	29.145	5.741	33	120.224	8.069	58	211.302	16.585	83	302.381	2.190
9	32.788	5.572	34	123.867	7.192	59	214.945	12.443	84	306.024	2.123
10	36.431	5.547	35	127.51	7.630	60	218.589	9.701	85	309.667	2.061
11	40.075	6.578	36	131.153	8.721	61	222.232	8.142	86	313.310	2.003
12	43.718	8.456	37	134.796	9.858	62	225.875	7.254	87	316.953	1.948
13	47.361	9.935	38	138.439	10.601	63	229.518	6.554	88	320.597	1.897
14	51.004	10.056	39	142.083	10.599	64	233.161	5.910	89	324.240	1.848
15	54.647	9.147	40	145.726	10.123	65	236.804	5.362	90	327.883	1.803
16	58.29	8.002	41	149.369	10.344	66	240.447	4.932	91	331.526	1.761
17	61.933	7.379	42	153.012	12.335	67	244.091	4.586	92	335.169	1.721
18	65.577	8.038	43	156.655	15.285	68	247.734	4.287	93	338.812	1.685
19	69.22	10.582	44	160.298	16.939	69	251.377	4.020	94	342.455	1.650
20	72.863	14.242	45	163.941	16.095	70	255.020	3.785	95	346.099	1.619
21	76.506	17.279	46	167.585	14.735	71	258.663	3.578	96	349.742	1.593
22	80.149	18.380	47	171.228	15.279	72	262.306	3.395	97	353.385	1.573
23	83.792	17.698	48	174.871	19.071	73	265.949	3.230	98	357.028	1.552
24	87.435	15.808	49	178.514	26.827	74	269.593	3.081	99	360.671	1.530
25	91.079	13.623	50	182.157	38.225	75	273.236	2.945			

S3 Hamiltonian of Model B

Here we present the explicit form of the Hamiltonian underlying model B and the respective parameters entering this model. The Hamiltonian $\hat{H}$ combines electronic and electron-phonon terms,

$$\hat{H} = \hat{H}_{\mathrm{el}} + \hat{H}_{\mathrm{e-ph}} + \hat{H}_{\mathrm{ph}} \tag{S3}$$

where $\hat{H}_{\mathrm{ph}}$ is given as

$$\hat{H}_{\mathrm{ph}} = \sum_{l=1}^{N_{\mathrm{F}}} \omega_l^{\mathrm{F}} \hat{b}_{\mathrm{F},l}^{\dagger} \hat{b}_{\mathrm{F},l} + \sum_{n=1}^{N} \sum_{l=1}^{N_{\mathrm{OT}}} \omega_l^{\mathrm{OT}} \hat{b}_{\mathrm{OT},n,l}^{\dagger} \hat{b}_{\mathrm{OT},n,l} + \omega_R \hat{b}_R^{\dagger} \hat{b}_R \tag{S4}$$

where $N = 13$ refers to the number of OT fragments, and $N_{\mathrm{F}} = N_{\mathrm{OT}} = 8$ effective modes are considered per fragment. The electronic part combines diagonal on-site energies and off-diagonal electronic coupling terms,

$$\hat{H}_{\mathrm{el}} = \hat{H}_{\mathrm{el}}^0 + \hat{H}_{\mathrm{coup}} \tag{S5}$$

and specifically

$$\begin{aligned} \hat{H}_{\mathrm{el}}^0 &= \epsilon^{\mathrm{LE}} \sum_{n=1}^{N} |\mathrm{LE}_n\rangle\langle \mathrm{LE}_n| + \sum_{n=1}^{N} \epsilon_n^{\mathrm{CS}} \sum_{n} |\mathrm{CS}_n\rangle\langle \mathrm{CS}_n| \\ \hat{H}_{\mathrm{coup}} &= J \sum_{n=1}^{N-1} (|\mathrm{LE}_n\rangle\langle \mathrm{LE}_{n+1}| + h.c.) + t \sum_{n=1}^{N-1} (|\mathrm{CS}_n\rangle\langle \mathrm{CS}_{n+1}| + h.c.) \\ &\quad + \lambda(|\mathrm{LE}_1\rangle\langle \mathrm{CS}_1| + h.c., \end{aligned} \tag{S6}$$

here $\epsilon^{\mathrm{LE}} = 100$ meV, $J = 100$ meV, $t = -120$ meV, $\lambda = -200$ meV and the ϵ_n^{CS} can be found in Table S2. Finally, the electron-phonon (vibronic) coupling part of the Hamiltonian is given in terms of Linear Vibronic Coupling (LVC) terms for the fullerene and thiophene

Table S2: On-site energies of the CS states used in model B.

n	ϵ_n^{CS} [meV]
1	0.0
2	33.6
3	47.4
4	56.0
5	61.8
6	65.7
7	68.4
8	70.0
9	70.9
10	71.2
11	71.1
12	70.5
13	69.5

parts, plus a single intermolecular mode R:

$$\hat{H}_{\mathrm{e-ph}} = \hat{H}^{\mathrm{F}}_{\mathrm{e-ph}} + \hat{H}^{\mathrm{OT}}_{\mathrm{e-ph}} + \hat{H}^{R}_{\mathrm{e-ph}}$$

where the individual components are given as

(i) vibronic coupling for N_{F} fullerene super-particle modes,

$$\hat{H}^{\mathrm{F}}_{\mathrm{e-ph}} = \sum_{l=1}^{N_{\mathrm{F}}} \left[\sum_{n=1}^{N} g_l^{\mathrm{F}} \left[\hat{b}^{\dagger}_{\mathrm{F},l} + \hat{b}_{\mathrm{F},l} \right] |\mathrm{CS}_n\rangle\langle\mathrm{CS}_n| \right] \tag{S7}$$

(ii) for $N \times N_{\mathrm{OT}}$ oligothiophene modes,

$$\begin{aligned} \hat{H}^{\mathrm{OT}}_{\mathrm{e-ph}} = & \sum_{n=1}^{N} \sum_{l=1}^{N_{\mathrm{OT}}} \Big[g^{\mathrm{OT}}_{\mathrm{CS},l} \left[\hat{b}^{\dagger}_{\mathrm{OT},n,l} + \hat{b}_{\mathrm{OT},n,l} \right] |\mathrm{CS}_n\rangle\langle\mathrm{CS}_n| \\ & + g^{\mathrm{OT}}_{\mathrm{LE},l} \left[\hat{b}^{\dagger}_{\mathrm{OT},n,l} + \hat{b}_{\mathrm{OT},n,l} \right] |\mathrm{LE}_n\rangle\langle\mathrm{LE}_n| \Big] \end{aligned} \tag{S8}$$

Here, it is assumed that the OT electron-phonon couplings are identical for all fragments (such that the vibronic couplings $g^{\mathrm{OT}}_{\mathrm{CS},l}$ and $g^{\mathrm{OT}}_{\mathrm{LE},l}$ do not carry a site index n).

(iii) for a single intermolecular mode R exhibiting both diagonal and off-diagonal couplings to the $|\mathrm{CS}_1\rangle$ and $|\mathrm{LE}_1\rangle$ states:

$$\hat{H}^R_{\mathrm{e-ph}} = g^R_{\mathrm{CS}_1,\mathrm{CS}_1}\left[\hat{b}^\dagger_R + \hat{b}_R\right]|\mathrm{CS}_1\rangle\langle\mathrm{CS}_1| + g^R_{\mathrm{LE}_1,\mathrm{CS}_1}\left[\hat{b}^\dagger_R + \hat{b}_R\right](|\mathrm{LE}_1\rangle\langle\mathrm{CS}_1| + h.c.), \quad \text{(S9)}$$

with $g^R_{\mathrm{LE,CS}} = -10/\sqrt{2}$ meV and $g^R_{\mathrm{CS}_1,\mathrm{CS}_1} = 30/\sqrt{2}$ meV, the same as in model A.

Table S3: Frequencies and linear coupling constants used in Model B. All quantities in meV.

l	ω_l^{F}	ω_l^{OT}	g_l^F	$g_{\mathrm{CS},l}^{\mathrm{OT}}$	$g_{\mathrm{LE},l}^{\mathrm{OT}}$
1	200.025	401.283	45.246	7.017	4.035
2	184.269	397.773	65.701	-0.077	2.921
3	177.853	182.714	-40.280	-67.849	-129.712
4	141.11	178.531	-17.511	57.668	46.885
5	93.952	134.550	28.026	-40.145	-32.908
6	79.933	111.848	-13.629	11.68	36.591
7	55.892	42.621	-23.732	-10.784	-20.211
8	33.264	18.316	9.86	-12.309	-7.77

S4 Convergence Tests for Model A

We carefully perform convergence checks of the numerical parameters of the MPS calculation. As discussed before, the most critical ones are the maximally allowed bond dimension during the time evolution and the maximum number of oscillator states taking into account. As visible in Fig. S2, we find for both quantities only numerically minor differences, indicating that the compared values are already close to convergence for model A. After these convergence tests, we settled at values $N_b^+ = 18$ and $D_{\max} = 300$ for the comparison to the ML-MCTDH calculation. This amounts to taking into account between 19 and 31 oscillator states for each mode in model A.

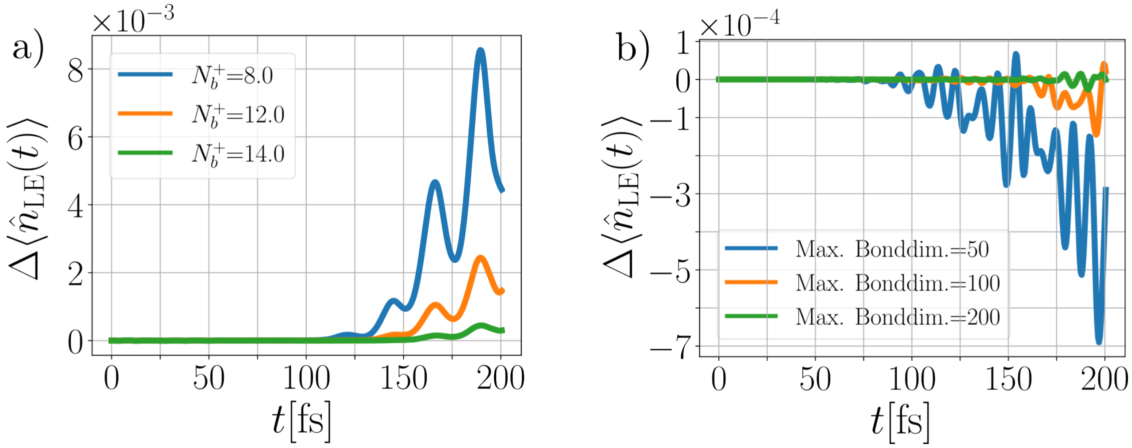

Figure S2: Difference of the LE density over time for the MPS calculation computed with different numerical parameters. Figure (a) depicts the difference in the local exciton density for different values of N_b^+ relative to $N_b^+ = 18$ for $D_{\max} = 300$ to check for the convergence in the oscillator states. In subfigure (b) we display the difference in the local exciton density for different values of $D_{\max}$ relative to $D_{\max} = 300$ for $N_b^+ = 14$ to check for the convergence in the bond dimension. Both plots indicate, that the MPS approach is close to convergence

The ML-MCTDH calculation, due to its different network structure, has a different set of numerical parameters than the MPS, that may impact the result of the dynamics. These include the number of SPFs in every layer, or a different tree structure for the decomposition of the tensor product. As briefly mentioned in the main text, all SPFs within the multilayer tree are optimized. The relevant multi-layer tree can be found in Fig. 4 of the main text. In

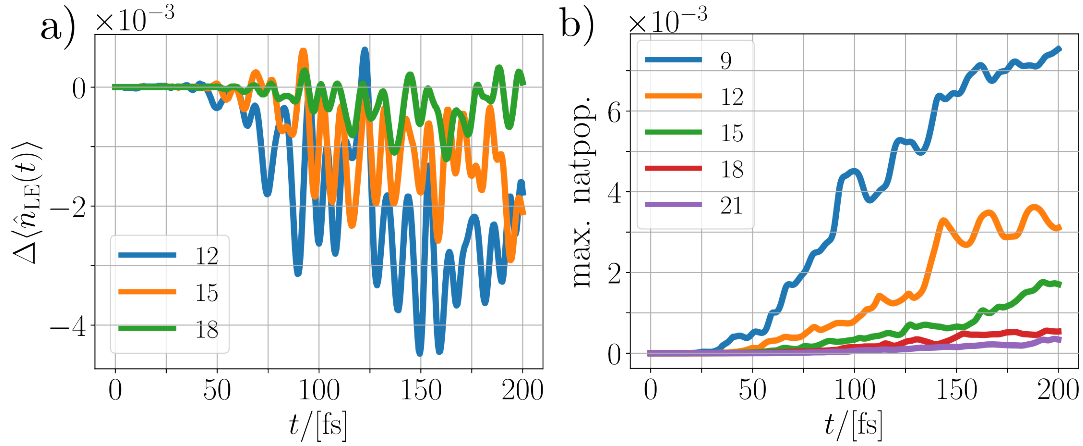

Figure S3: (a) shows the difference of the LE density over time for the ML-MCTDH calculation computed with different number of SPF in the tree, relative to the best result with 21 SPFs in the top layer. The labels correspond to the highest amount of SPFs in the top layer, exluding the electronic degree of freedom.. In (b) we depict the maximum natural population of the highest natural orbital of the ML-MCTDH calculation over time. Both plots indicate, that the ML-MCTDH calculation is close to convergence, with a maximum natural orbital population around 5×10^{-4}.

Fig. S3 (a) we compare the difference of the LE density dynamics over time for a different number of SPFs in the tree, relative to the best result with 21 SPFs in the top layer. The labels correspond to the highest amount of SPFs in the top layer, exluding the electronic degree of freedom. We observe a decreasing deviation, on the same order of magnitude of the MPS convergence check, as the number of SPFs is increased, as a sign for convergence. Alternatively, we study the maximum natural populations, which is an alternative approach to check on the quality of the result[1,2]. Also for this quality check, which is depicted in Fig. S3 (b), we find, with maximum natural orbital populations below 5×10^{-4} until the end of the dynamics, very good results.

S5 Convergence Tests of Model B

Before we again tackle the comparison between the two methods let us ensure the validity of the MPS results by performing convergence checks of the technical simulation parameters. We first study the convergence of n_{LE1} with the number of basis states in the local Hilbert spaces of the modes. For this, Eq. 8 of the main text introduces the numerical convergence parameter N_b^+. To test the convergence with respect to this parameter we plot plot in Fig. S4 (a) the difference of the LE$_1$ population over time with respect to the simulations with $N_b^+ = 18$. The curves indicate that up to a time scale of about 35 fs, the dynamics is essentially the same for all the considered N_b^+s. After this time, however, deviations in n_{LE1} start to appear, which occur as oscillations around zero with magnitudes on the order of few times 10^{-3} and which are similar for all the considered cases. In our impression, these small oscillations are a result of the interplay of a fixed maximum bond dimension and a slightly different configurational space, due to the different number of basis states in each factor space. As a result, these deviations do not grow in magnitude over time. As the amplitudes of these oscillations are also of similar magnitude as the result from model A [cf. Fig. S2 (a)], we regard the dynamics to be sufficiently converged with respect to N_b^+.

In the second test we compare the difference of the exciton density in the LE$_1$ state for three different maximally allowed bond dimensions $D_{\max}$ relative to $D_{\max} = 400$. The result of this comparison is summarized in Fig. S4 (b). We find that, similar to the first test, the difference remains very small for small times. This is not surprising because the entanglement between the different degrees of freedom has to build up during the evolution, which requires some minimum time. However, after this time scale we find increasing deviations to the reference on the order of 0.01 and, hence, orders of magnitude larger than those encountered in the case of model A [cf. Fig. S2 (b)]. This observation already hints at a significantly stronger entanglement between the individual degrees of freedom than in model A. To study this in more detail, we study the maximum bond dimension of the MPS over time, which is defined as the maximum rank of the matrices appearing in Eq. 6 of the main text, as a

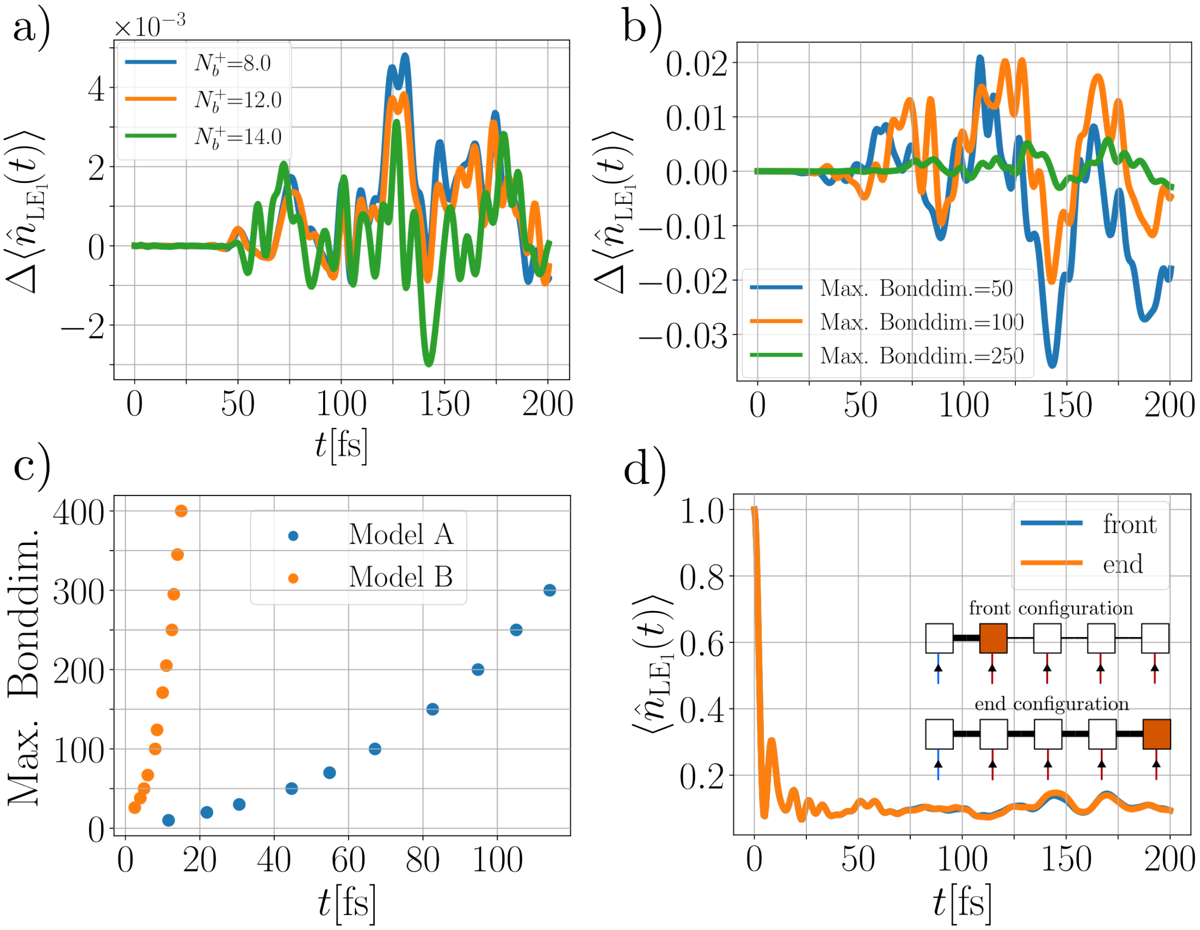

Figure S4: Difference of the LE_1 density over time for the MPS calculation computed with different numerical parameters. (a) displays the difference in the LE_1 density for different values of N_b^+ relative to $N_b^+ = 18$ for $D_{\max} = 250$. Panel (b) shows the difference in the local exciton LE_1 density for different values of $D_{\max}$ relative to $D_{\max} = 400$ for $N_b^+ = 18$ to check for the convergence in the bond dimension. (c) compares the maximum bond dimension over time for model A and model B. Although both show an exponential increase, the bond dimension in model B increases very quickly, reaching large values within a few fs. Panel (d) provides a comparison of the LE_1 density computed with two different ordering (intermolecular mode at beginning/end) of the local Hilbert spaces given in the inset for $N_b^+ = 14$ and $D_{\max} = 250$. This shows, that the ordering of the local Hilbert spaces in the MPS calulation plays only a minor role.

proxy for the entanglement in Fig. S4 (c). We find that the maximum bond dimension of the MPS increases rapidly, reaching the value of 400 already within 20 fs, which confirms the hypothesis. To keep the calculations feasible we restrict the maximum bond dimension to 400 and use N_b^+ =18. This is justified because the deviations of the LE_1 density to the reference calculation [cf. Fig. S4 (b)] are below 0.005 over the simulation time.

To test, how strongly the ordering of the local Hilbert spaces in the MPS calculation impacts the result, we compare the dynamics of the LE_1 density for the ordering of the local Hilbert space of the intermolecular mode either directly at the electronic factor space (termed "front") or the very end of the chain (termed "end"). In the first case, the intermolecular mode can directly communicate to the electronic factor space, whereas in the second case it has to communicate over the factor spaces of all the other modes, which potentially requires a larger bond dimension. However, as depicted in Fig. S4 (d), we find only minor differences, which indicates, that the ordering does not significantly impact the dynamics and we are close to a converged result.

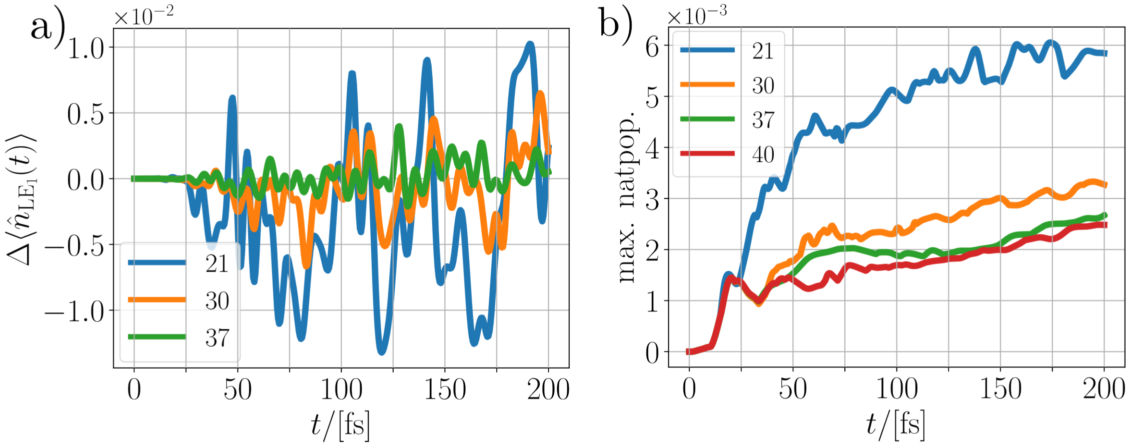

Figure S5: In (a) we depict the difference of the LE_1 density over time for the ML-MCTDH calculation computed with different SPFs in the tree, relative to the best result with 40 SPFs in the top layer. The labels correspond to the highest amount of SPFs in the top layer, excluding the electronic degree of freedom. Panel (b) shows the maximum natural orbital population of the ML-MCTDH tree versus time. The value of around 2×10^{-3} is very satisfactory.

For the ML-MCTDH approach, we perform the convergence checks analogously to model

A. That is, we tune the number of SPFs in all layers and study the changes in the population dynamics of the LE_1 state. The relevant multilayer tree is depicted in Fig. 10 of the main text. The difference of the LE_1 population dynamics for different number of SPFs relative to the best result with 40 SPFs in the top layer is depicted in Fig. S5 (a). We find a decrease in the difference as an indication for convergence. We find significantly larger deviations compared to model A, consistent in order of magnitude with the results of the convergence checks done in the MPS approach. As a different measure of convergence we again study the maximum natural orbital populations. For the maximum natural orbital populations we also observe convergence, but as for the density dynamics test, the deviations are orders of magnitude larger than in model A. Still, for the significantly larger system the maximum natural orbital populations of 2×10^{-3} until the end of the dynamics can be regarded as a satisfactory result.

S6 Detailed Comparison of State Populations

In this section, a more detailed view is given of the dynamical results for model B, with some emphasis on the generation of free carriers. To this end, the population of the higher CS_n states is relevant. Additionally we compare the results of the ML-MCTDH calculation to additional calculations based on a different tree structure (denoted by T2). This alternative tree structure is depicted in Fig. S9. Complementary to Figs. 11 and 12 in the main text, Fig. S6 shows the population of all 13 CS states for both MPS and ML-MCTDH.

Rather than looking at individual populations, one can also divide the CS manifold into those states that "precede the maximum of the Coulomb barrier" (CS_1 to CS_6) and a second subset of states (CS_7 to CS_{13}) "beyond the maximum of the Coulomb barrier". Summing up the populations of these subsets can loosely be seen as the populations of "bound" and "free" charge carriers, respectively. The corresponding populations are shown in Fig. S7. Here, again, quantitative deviations between MPS and ML-MCTDH can be seen. Deviations start to set in as early as about 60 fs.

We note that similar deviations to those of the CS populations can be seen for the LE populations in Fig. S8.

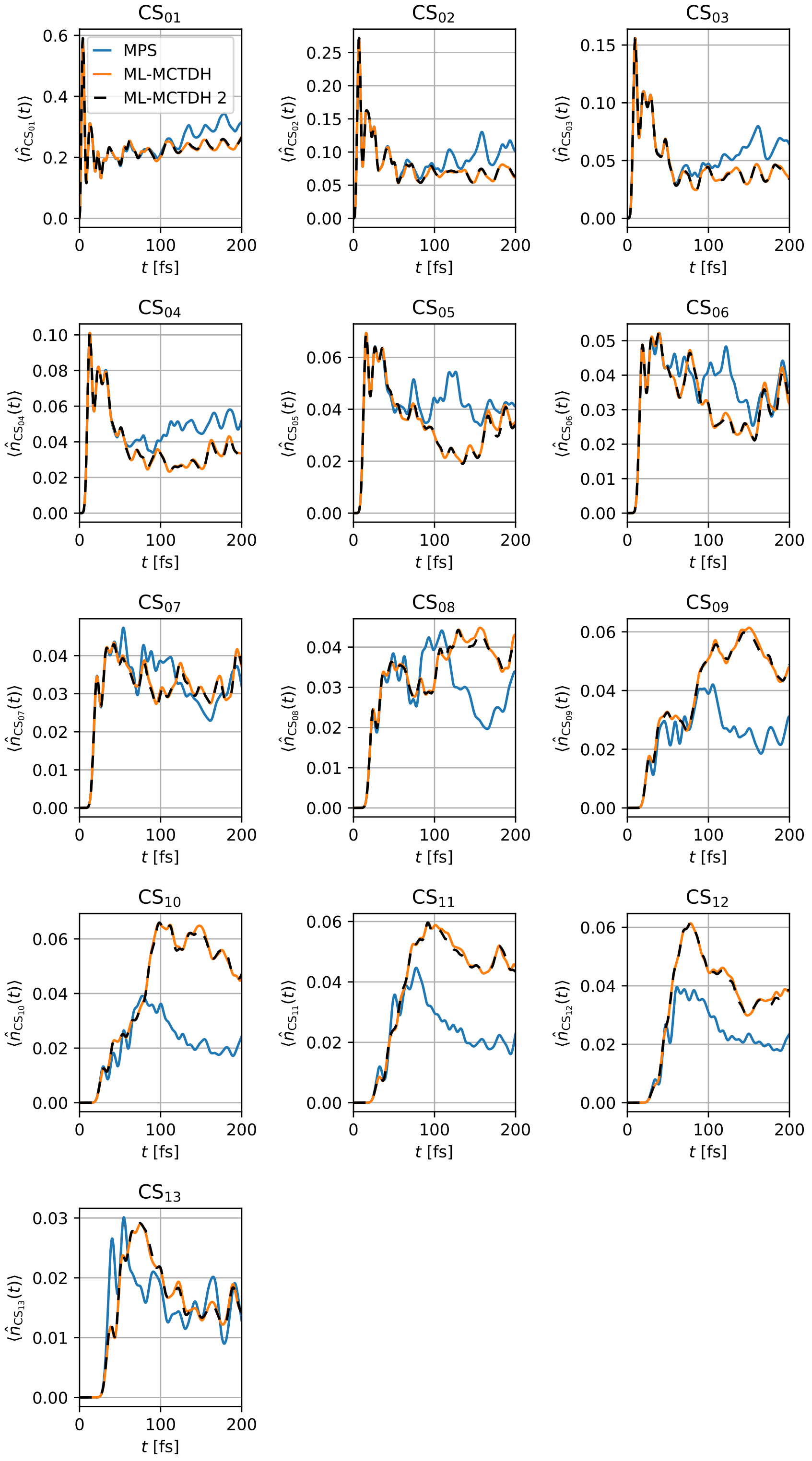

Figure S6: Population of all 13 CS states for MPS and ML-MCTDH for the two different tree structures.

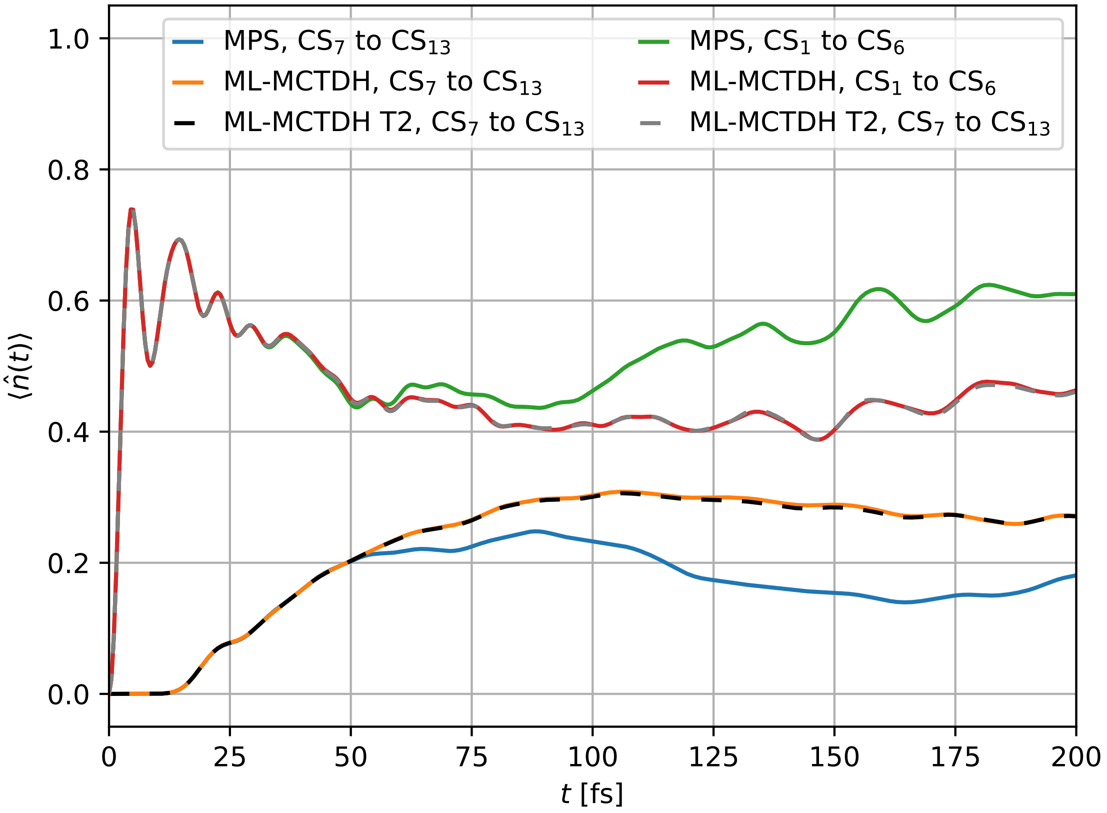

Figure S7: CS populations for "bound" (CS_1 to CS_6) and "free" (CS_7 to CS_{13}) charge carriers.

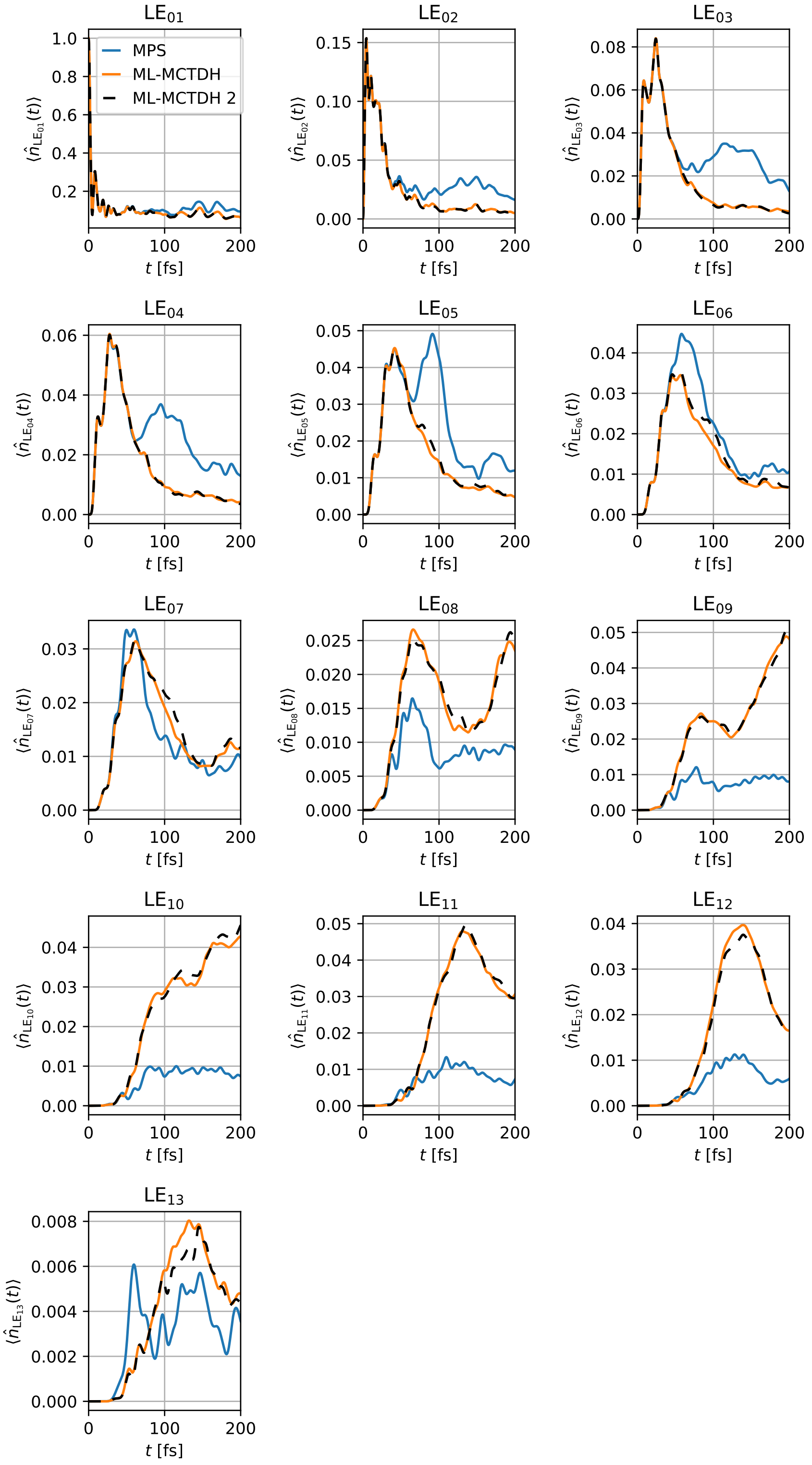

Figure S8: Population of all 13 LE states for MPS and ML-MCTDH for the two different tree structures.

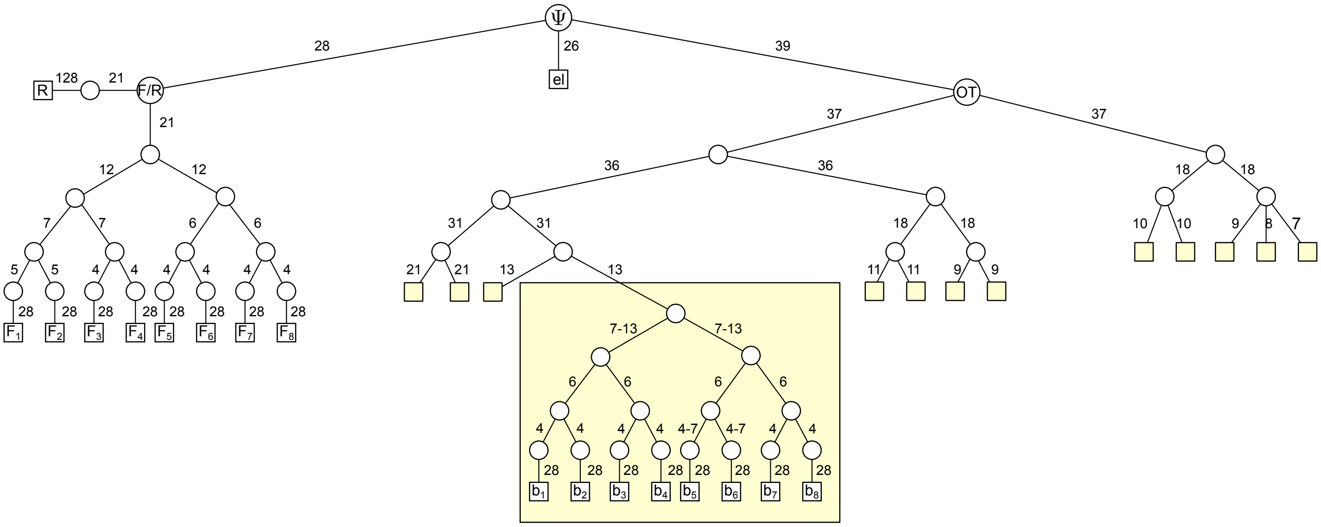

Figure S9: Alternative ML-MCTDH tree structure (T2) used for the comparison of the populations shown in Figs. S6-S8. Differently from the multi-layer tree depicted in Fig. 10 of the main text, the present variant does not distinguish between different groups of oligothiophene modes (i.e., up to 1500 cm^{-1} and over 3000 cm^{-1}. The performance and results obtained with this alternative tree structure are very similar to the reference tree shown in the main text.

References

(1) Manthe, U.; Meyer, H.; Cederbaum, L. S. Wave-packet dynamics within the multiconfiguration Hartree framework: General aspects and application to NOCl. *The Journal of Chemical Physics* **1992**, *97*, 3199–3213.

(2) Beck, M. H.; Jäckle, A.; Worth, G. A.; Meyer, H. The multiconfiguration time-dependent Hartree (MCTDH) method: a highly efficient algorithm for propagating wavepackets. *Phys. Rep.* **2000**, *324*, 1–105.
